# Comparative transcriptome analysis indicates conversion of stamens into pistil-like structures in male sterile wheat (*Triticum aestivum* L.) with *Aegilops crassa* cytoplasm

**DOI:** 10.1186/s12864-020-6450-2

**Published:** 2020-02-04

**Authors:** Qi Liu, Zihan Liu, Wei Li, Xiyue Song

**Affiliations:** College of Agronomy, Northwest A & F University, Yangling, 712100 China

**Keywords:** Anther transcriptome, Cytoplasmic male sterility, Hybrid wheat, Pistil-like

## Abstract

**Background:**

*Aegilops crassa* cytoplasm is an important source for investigating cytoplasmic male sterility (CMS). Moreover, the stamens of line C303A exhibit a high degree of pistillody, turning almost white. However, the molecular mechanism that underlies pistillody in C303A remains unclear. Therefore, to obtain a better understanding of pistillody in C303A, the phenotypic and cytological features of C303A were observed to identify the key stage for the homeotic transformation of stamens into pistil-like structures. Transcriptome profiles were determined for stamens using Illumina RNA sequencing.

**Results:**

Morphological observations of the CMS wheat line with *Aegilops crassa* cytoplasm C303A showed that the pistils developed normally, but the stamens were ultimately aborted and they released no pollen when mature. According to paraffin section observations, the stamens began to transform into pistils or pistil-like structures in the binucleate stage (BNS). Therefore, the stamens were collected from line C303A and its maintainer 303B in the BNS for transcriptome sequencing. In total, 20,444 wheat genes were determined as differentially expressed in C303A and 303B stamens, with 10,283 upregulated and 10,161 downregulated genes. Gene Ontology enrichment analyses showed that most of the differentially expressed genes (DEGs) were annotated with GO terms comprising metabolic process, cell, cellular process, catalytic activity, and cell part. Analysis based on the Kyoto Encyclopedia of Genes and Genomes database showed that the enriched DEGs were mainly associated with energy metabolism. We also found several essential genes that may contribute to pistillody in C303A. These findings suggest that disrupted energy metabolism and reactive oxygen metabolism induce pistillody and eventually lead to abortion in C303A.

**Conclusion:**

We determined the complex transcriptome profiles for C303A stamens and demonstrated that disrupted energy metabolism and class B MADS-box genes are related to pistillody. These findings may facilitate future studies of the mechanistic response of the wheat stamen and pollen development in CMS**.**

## Background

Wheat is a staple food for 35% of the world’s population [1] and the second largest staple food crop after rice, with around 220 million ha cultivated worldwide [2]. China is the largest producer and consumer of wheat throughout the world, with a cultivation area of about 24 million ha and an average yield of 4762 kg ha^− 1^. Many significant challenges are affecting China such as an increasing population size and reduced arable land area. Therefore, improving the grain yield is an inevitable requirement for ensuring food security [3]. Indeed, increasing the wheat yield is a long-term goal of wheat breeding and the utilization of heterosis is the best method for increasing yields and satisfying global food safety requirements for crops such as maize, rape, sunflower, rice, and sorghum [4]. Moreover, male sterile plants are crucial breeding tools for harnessing hybrid vigor or heterosis in hybrid crops, and they also provide valuable materials for studying stamen and pollen development as well as nuclear–cytoplasmic interactions [5].

*Aegilops crassa* cytoplasm is a vital source of cytoplasmic male sterility (CMS), which has no harmful effects on the agronomic characteristics of common wheat. Line C303A with cytoplasm from *Ae. crassa* is an outstanding wheat germplasm resource for CMS, but the problem with C303A is that similar to the complex restoration of fertility, it exhibits poor outcrossing in wheat with few restorer lines. Thus, heterosis is difficult to apply in C303A, although it has great potential for research as a germplasm resource. In particular, the stamens of C303A exhibit a high degree of pistillody, where they are almost white compared with the maintainer line 303B because of nuclear–cytoplasmic interactions. A previous study identified an alloplasmic line comprising Norin 26 (N26) with *Ae. crassa* cytoplasm, which exhibits male sterility under long-day conditions (> 15 h light period) because of pistillody [6]. Thus, we assume that certain factors in the *Ae. crassa* cytoplasm promote pistillody and they may be responsible for feedback regulation of nuclear genes [7]. However, there have been few in-depth studies of pistillody in wheat. C303A is a useful material for studying pistillody in wheat, so we investigated some of the key characteristics of C303A, including its floret morphology, cytological mechanism, physiological indexes, and the molecular mechanism associated with pistillody.

The analysis of mutations in *Arabidopsis thaliana* and *Antirrhinum majus* allowed the formulation of the ABC model of flower organs [8]. Subsequent studies that analyzed orthologs of the MADS-box genes provided novel insights into the ABC mechanisms, and a landmark in plant developmental biology is development of the ABCDE model of floral organ formation [9, 10]. In this model, classes B, C, and E specify stamens, class C and E genes define carpels, and class D and E genes determine ovules. The MADS-box genes cloned in wheat include 13 MIKC c-type MADS-box subfamily genes. Two PISTILLATA (PI)-type class-B MADS-box genes were isolated from (cr)-CSdt7BS, i.e., *WPI2* and *WPI1*, and it was suggested that pistillody in (cr)-CSdt7BS wheat is caused by alterations to the expression patterns of class-B MADS-box genes [11, 12]. Moreover, loss of function by class B MADS-box genes leads to pistillody in *Arabidopsis* [13] and *Antirrhinum* [14]. Therefore, the class B MADS-box genes could be related to the induction of pistillody in wheat.

The molecular mechanisms responsible for stamen development and nuclear–cytoplasmic interactions are clearly understood in *Arabidopsis thaliana*, rice, maize, and other model plants, but they have rarely been studied in non-model plants. Wheat is a heterologous hexaploid plant with a complex genetic background, and it is inefficient and difficult to study the large number of genes involved in stamen development and the nuclear–cytoplasmic interaction network using traditional molecular biology methods. However, high-throughput RNA transcriptome sequencing (RNA-Seq) can provide comprehensive and rapid access to most of the transcript information for wheat stamens during the binucleate stage (BNS), thereby facilitating systematic analysis of the differentially expressed genes (DEGs) related to pistillody. In the present study, RNA-Seq was conducted using the stamens from C303A and its maintainer line 303B in order to identify the metabolic processes and transcription factors that might contribute to pistillody in C303A. A comprehensive understanding of the changes in the genetic network and expression patterns associated with pistillody in C303A will enable future applications of the molecular mechanisms involved with stamen development and nuclear–cytoplasmic interactions.

## Results

### Phenotypic characteristics of C303A and 303B stamens

**Fig. 1:**
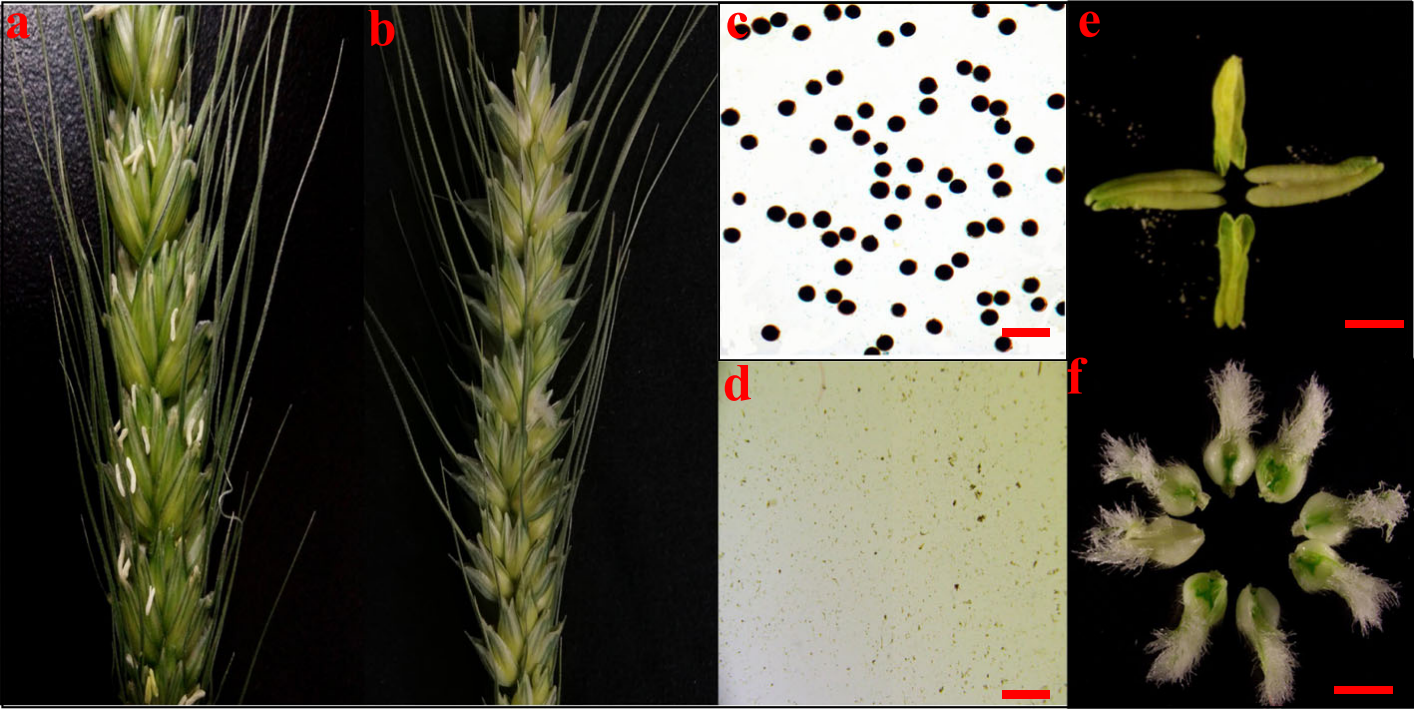
Morphology of 303B (**a**, **c**, **e**) and C303A (**b**, **d**, **f**) plants. **a**, **b** Inflorescences of 303B and C303A, showing stamens. **c**, **d** Microspores identified by I_2_–KI staining. **e**, **f** Morphology of 303B and C303A stamens during the trinucleate stage. Scale bars represent 50 μm (**c**, **d**), and 200 μm (**e**, **f**)

**Fig. 2:**
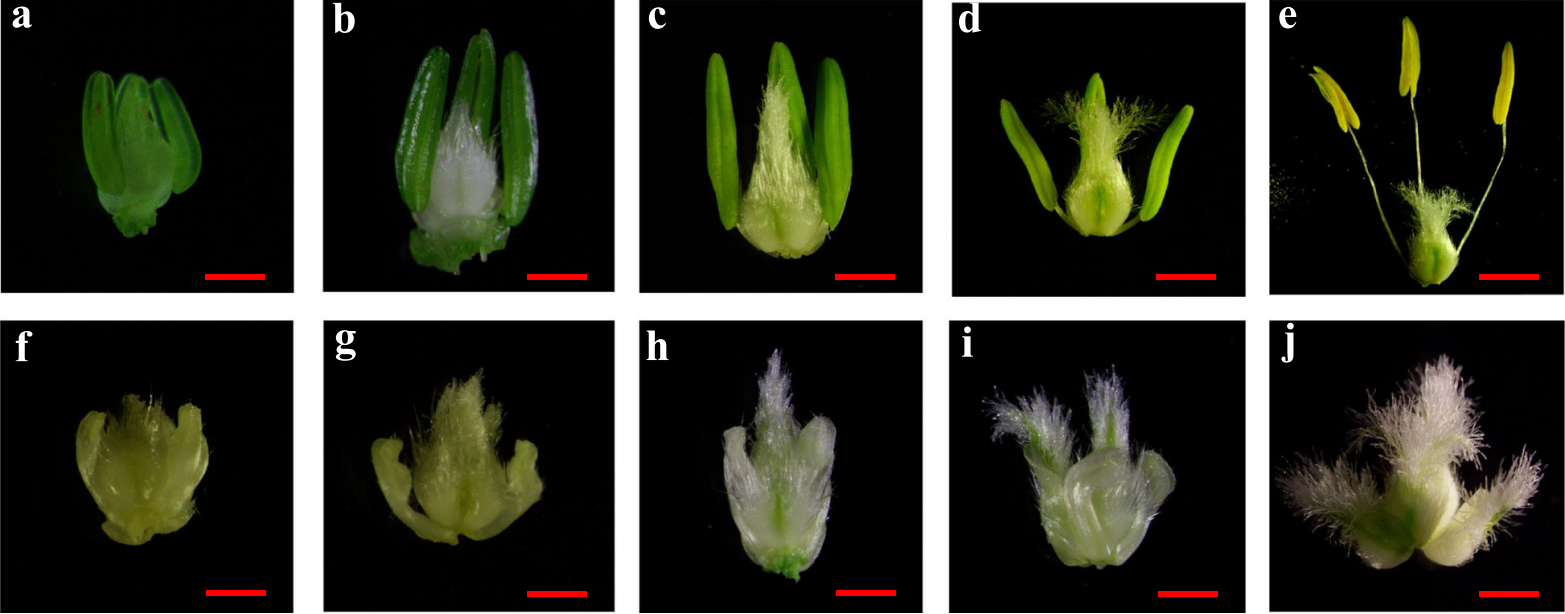
Comparisons of the anther phenotypes in 303B (**a**–**e**) and C303A (**f**–**j**). **a**, **f** TDS, tetrad stage; (**b**, **g**) EUNS, early uninucleate stage; (**c**, **h**) LUNS, late uninucleate stage; (**d**, **i**) BNS, binucleate stage; and (**e**, **j**) TNS, trinucleate stage. Scale bars represent 1 mm in (**a**–**j**)

**Fig. 3:**
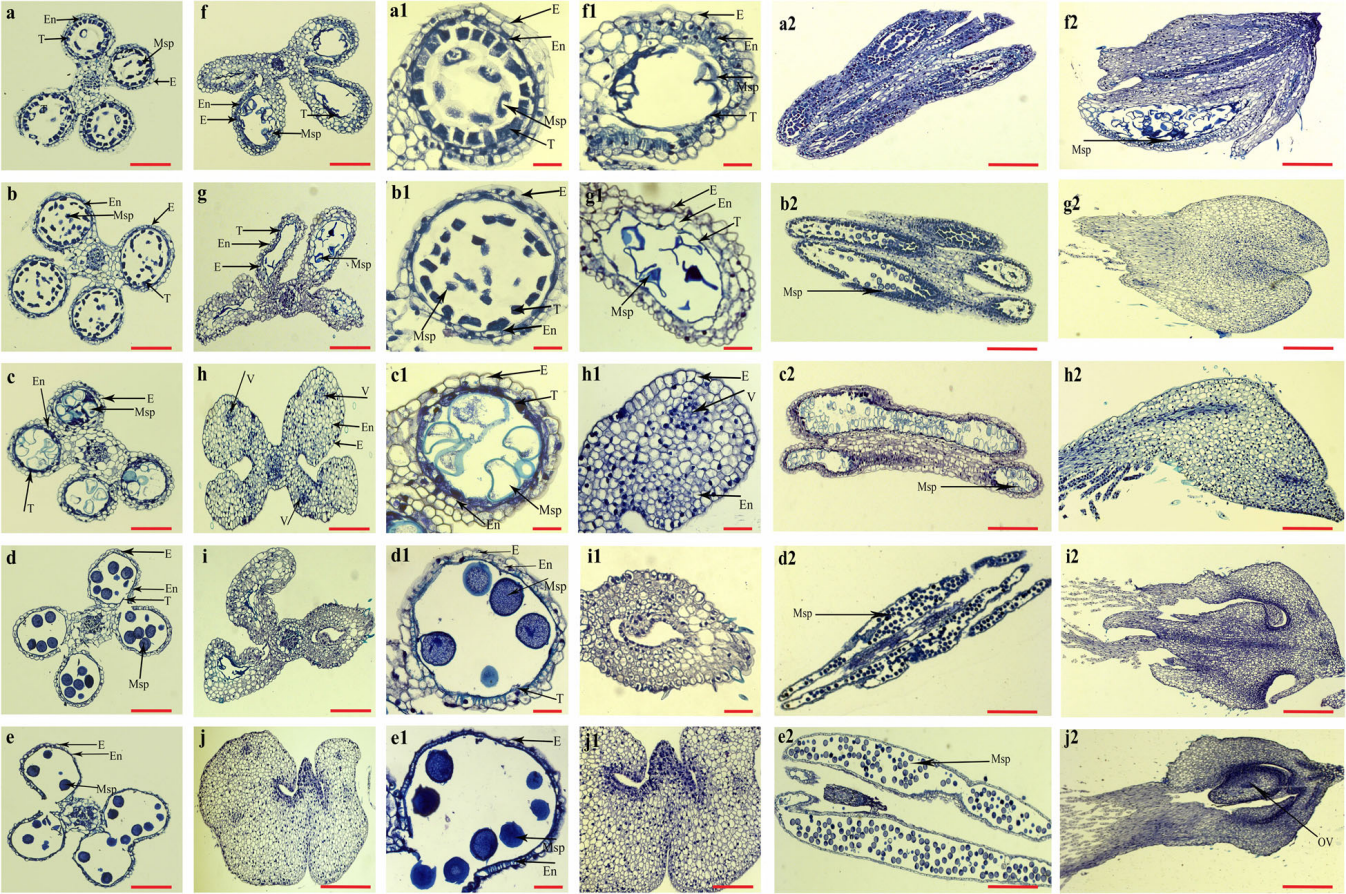
Transverse and longitudinal sections of normal and pistil-like stamens. Transverse sections of normal stamens from 303B (**a**–**e**) and pistil-like stamens from C303A (**f**–**j**). Comparisons of anther locules in 303B and C303A during different stages. Longitudinal sections of normal stamens from 303B (a2–e2) and pistil-like stamens from C303A (f2–j2). E: Epidermis; En: endothecium; ML: middle layer; T: tapetum; Msp: microspores. V: vascular bundle, Ov: ovule. Scale bars represent 100 μm in (**a**–**i**, j1), 50 μm (a1–i1), and 200 μm (a2–j2, **j**)

The wheat CMS line C303A was developed from stable sterile lines by consecutive backcrossing with 303B as the donor parent over 20 times in Yangling, China. To determine the abortive morphological features of C303A, we collected C303A stamens in five developmental stages. Comparative field observations showed that except for the floral organs, there were no significant differences in the growth and overall morphology of the CMS line C303A and its isomaintainer line 303B (Fig. 1). In the binucleate stage (BNS), the 303B anthers were quite full and yellowish, where the upper and lower ends were bifurcated and bright yellow, and normal cracking was accompanied by the release of a large amount of pollen in the trinucleate stage (TNS). By contrast, the C303A anthers were white in the tetrad stage. Stamen malformation occurred in the uninucleate stage and the individual stamens had curved folds. Some stamens were combinations of stamens and pistils in the BNS. The stamens were completely transformed into pistils in the TNS (Fig. 2). Moreover, we prepared paraffin sections to accurately observe the cytological structure of the stamens with pistillody. As shown in Fig. 3, there were clear differences between the fertile stamens and pistillody stamens. Compared with 303B in the tetrad stage, the four anther locules were shrunken and shriveled in C303A. In addition, marked degradation of one anther locule occurred with no microspores, and another anther locule was empty with only a very thin tapetum. However, a small number of microspores were detected in the other two anther locules. The degradation of the anther locules increased in the early uninucleate stage, and the microspores were shrunken and clearly condensed in another anther locule. During the later uninucleate stage, the outlines of the tapetal cell and microspores were totally invisible, and some vascular bundles were visible after the degradation of the anther locule, which contributed to the transport of nutrients to the locule for ovule formation. In the BNS, the two degenerate chambers began to merge and we consider that this may have been the start of the ovule formation process. The ovule structure in the pistillody stamens was determined based on transverse and longitudinal sections until the TNS. The results indicated that the pistillody stamens contained ovule structures, instead of pollen grains and tapetum, and thus the C303A stamens were unable to produce mature pollen grains that could be detected by potassium iodide staining. Thus, we deduced that pistillody might occur in the BNS.

### Sequence analysis using RNA-Seq

To understand the basic molecular mechanisms responsible for pistillody at the transcriptional level, we employed an Illumina HiSeq PE1500 sequencer for transcriptome sequencing analysis using the stamens from CMS line C303A and its maintainer line 303B in the BNS. Stamens were analyzed three times with a total of three biological replicates and the sequencing read lengths were 150 bp. After filtering out reads with > 10% ambiguous nucleotides, adapter sequences, and low-quality regions, 270,683,956 clean reads were obtained, with 131,000,548 reads from the maintainer line, and 139,683,408 from the CMS line. The GC content ranged from 55.80 to 59.63%, and the Q20 percentage exceeded 88.93%. The clean reads obtained from each sample were matched with the *Triticum aestivum* reference sequence, where the alignment efficiency ranged between 60.46 and 68.09% (Table 1). The throughput and sequencing quality showed that the RNA-Seq data were adequate for further analysis.

**Table 1:** Transcriptome-sequencing data quality and genome mapping

Groups	Total Reads	Clean Reads	GC (%)	N (%)	Q20 (%)	Total Mapped Reads	Mapping Ratio (%)
303B-1	39,676,066	37,327,088	57.9	0	88.93	10,072,351	60.46
303B-2	48,075,242	46,277,952	56.2	0	91.75	15,228,480	66.31
303B-3	49,378,238	47,395,508	57.84	0	91.43	12,332,368	62.92
C303A-1	46,684,522	45,850,716	59.63	0	92.72	13,072,169	60.75
C303A-2	47,100,488	45,722,288	56.09	0	91.27	14,683,259	64.74
C303A-3	49,194,222	48,110,404	55.8	0	92.87	15,363,865	68.09
Total	280,108,778	270,683,956	–	–	–	13,458,749	63.88

### Identification of DEGs by RNA-Seq

**Fig. 4:**
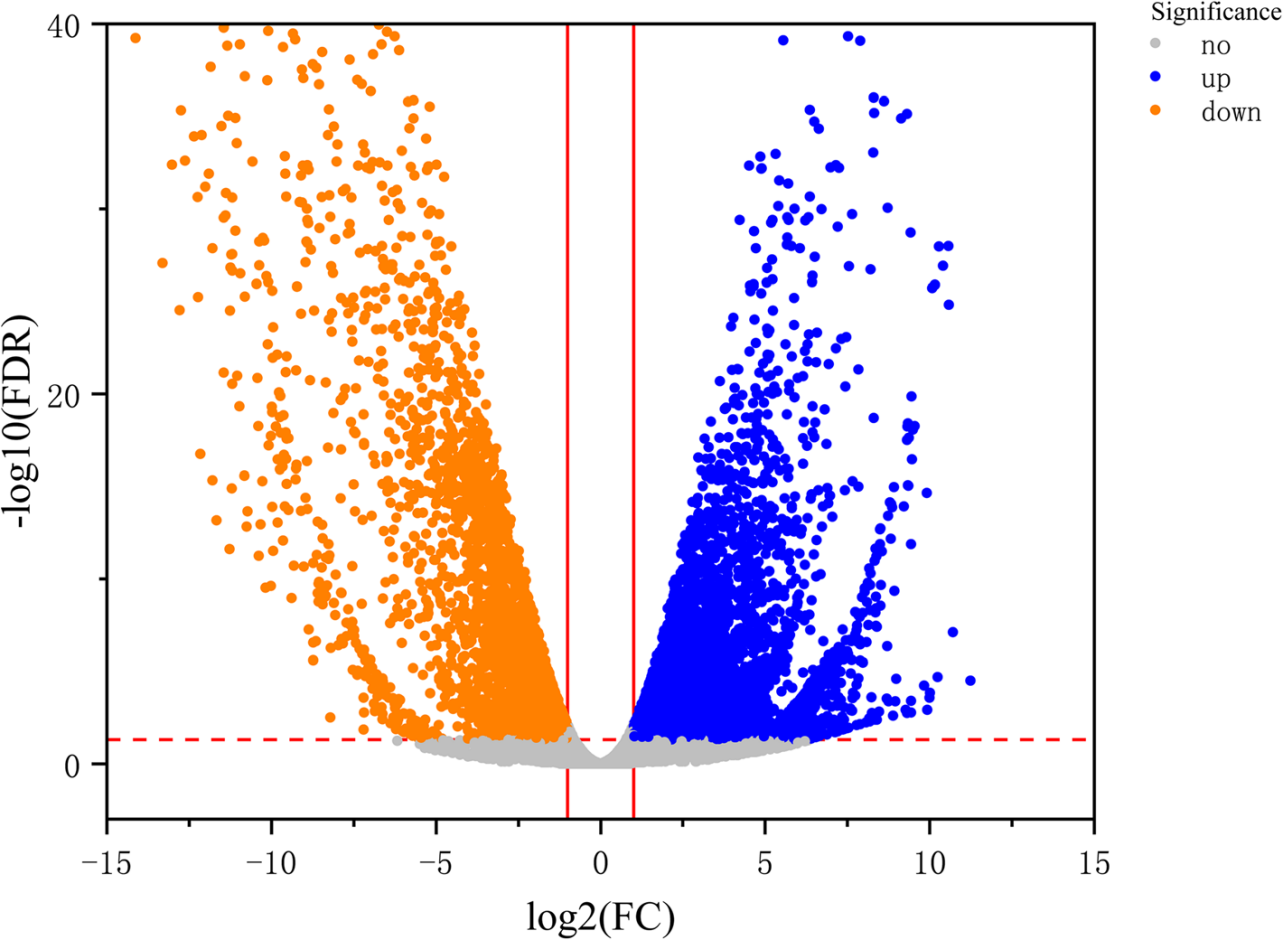
Comparison of gene expression levels in C303A relative to 303B during the binucleate stage. Gray dots indicate no significant difference. Blue dots indicate significantly upregulated genes. Orange dots indicate significantly downregulated genes

In total, RNA-Seq detected 179,898 genes. To determine significant differences in the gene expression levels, we used a false discovery rate (FDR) < 0.05 and log 2 Fold Change (|log 2 FC|) > 1) as the thresholds. The DEGs in C303A and 303B during the BNS were compared based on the significant differences (Fig. 4). In total, 20,444 genes were differentially expressed in C303A and 303B stamens. These DEGs comprised 10,283 upregulated genes and 10,161 downregulated genes in the C303A stamens compared with the 303B stamens.

### Gene ontology (GO) enrichment analyses of DEGs

**Fig. 5:**
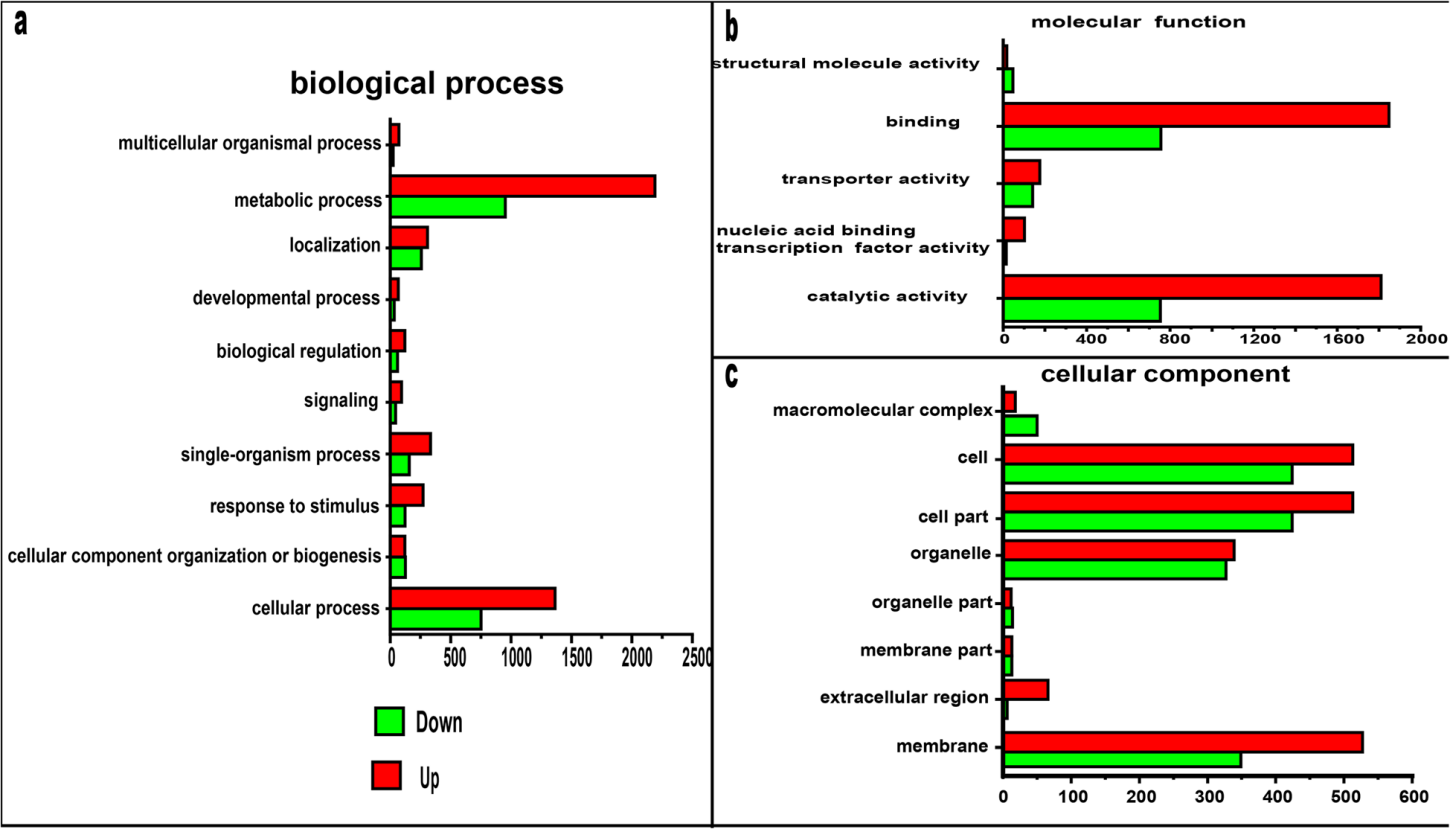
Gene Ontology (GO) classifications of differentially expressed genes (DEGs) in C303A and 303B during the binucleate stage

GO is a universal standardized gene functional classification system based on a dynamically updated controlled vocabulary and rigidly defined concepts for comprehensively describing the properties of genes and their products in organisms [15]. After enrichment analysis, the DEGs found in C303A were annotated according to 36 functional groups (Fig. 5). Among the biological process functions, the central DEGs were associated with cellular processes, single-organism process, localization, and metabolic processes. In terms of cellular components, the DEGs were associated with cell, cell parts, membranes, and organelles. In addition, binding, catalytic activity, and transporter activity were closely related to molecular functions. Twenty significantly enriched GO terms were found in the biological process functions according to hypergeometric tests. Among these 20 GO terms (Additional file 1: Table S1), the q-values equaled zero for two terms comprising carbohydrate metabolic process (GO: 0005975) and single-organism metabolic process (GO: 0044710). The DEGs found in different functional categories may provide valuable resources for studying stamen development in C303A.

**Fig. 6:**
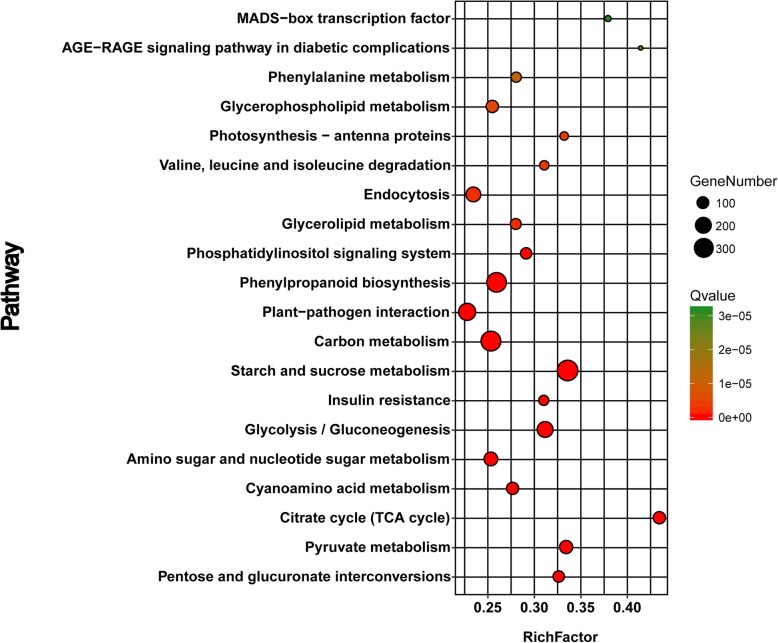
Top 20 significantly enriched pathways. “Large RichFactor” indicates a high degree of enrichment

In order to identify the biological pathways involved, the DEGs in the BNS were mapped to 129 pathways in the Kyoto Encyclopedia of Genes and Genomes (KEGG) database, where the top 36 pathways (Additional file 2: Table S2) were considered significant at a cut-off FDR corrected q-value <0.05. The main DEGs were associated with starch and sucrose metabolism, amino sugar and nucleotide sugar metabolism, glycolysis, phenylpropanoid biosynthesis, pyruvate metabolism, tricarboxylic acid cycle (TCA cycle), pentose and glucuronate interconversion, and cyanoamino acid metabolism (Fig. 6). The expression levels of most of the genes that mapped to the nine significantly enriched pathways tended to be downregulated, except for the circadian clock systems and phenylpropanoid biosynthesis pathways. Thus, starch and sucrose metabolism, glycolysis, and the TCA cycle may be essential for pollen development. Previous studies have also shown that the TCA cycle has a pivotal role in plant male sterility [16], and the normal energy metabolism processes in plants can support their growth and development. Therefore, we focused on the significant genes related to these pathways in order to explain why no microspores were present in the pistillody stamens.

### Identification of MADS-box transcription factor involved in pistillody

**Fig. 7:**
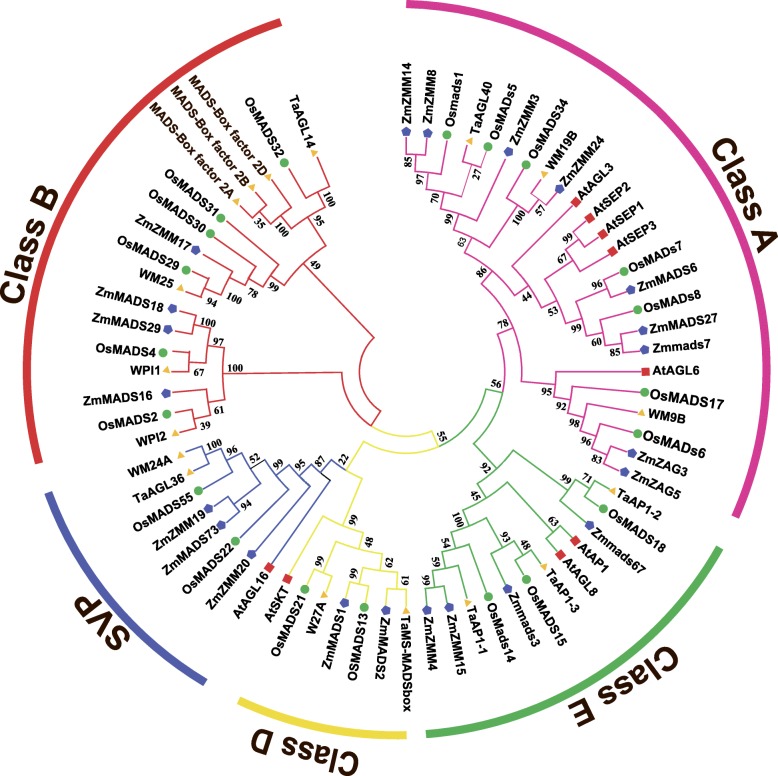
Phylogenetic tree constructed for MADS-box transcription factor genes in different plant species. The full-length amino acid sequences of MADS-box transcription factor genes from maize (*Zea mays*), *Arabidopsis* (*Arabidopsis thaliana*), rice (*Oryza sativa*), and wheat (*Triticum aestivum* L) were obtained from the online NCBI database. Phylogenetic trees were constructed using MEGA 6.0. (Detailed information for these genes is provided in Additional file 4: Table S4)

**Fig. 8:**
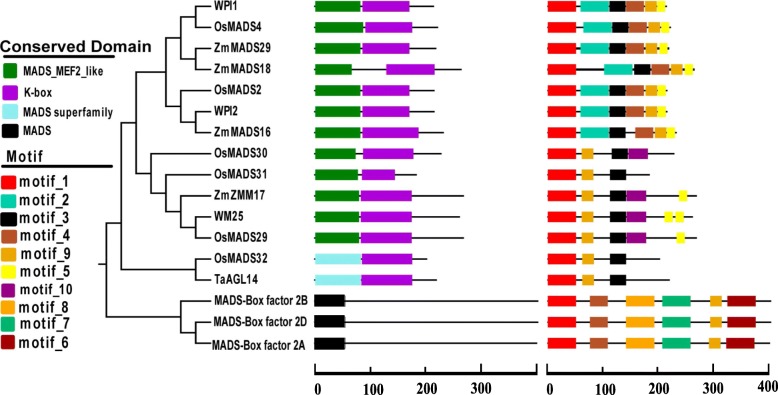
Phylogenetic relationships of conserved domains and motif compositions for class B MADS-box genes

In plants, members of the MADS-box gene family play essential roles in the ABCDE model of flowering. According to this model, three (*TaAGL40, WM19B,* and *WM9B*), one (*WM27A*), two (*WM24A* and *TaAGL36*), and five (*TaAGL14*, *WM25, MADS-Box factor 2A, MADS-Box factor 2D,* and *MADS-Box factor 2B*) wheat genes were assigned to class A, class D, SVP, and class B, respectively (Fig. 7). Previous studies also identified several class B MADS-box genes in wheat, such as *WPI2* and *WPI1*. These genes are mainly related to the transformation of wheat from vegetative growth to reproductive growth. The expression patterns of these genes differ in heterogeneous wheat and common wheat, which may contribute to the transformation of stamens into pistil-like structures. The conserved domains and motif compositions were analyzed for the class B MADS-box genes in the present study. As shown in Fig. 8, a conserved K-box is present in most of the class B MADS-box genes, except for the MADS-box factor 2 genes. A distinct MADS superfamily exclusively contains *OsMADS32* and *TaAGL14.* Analysis of the motif compositions identified 10 conserved motifs in the class B MADS-Box genes, which were designated as motif_1 to motif_10. Motif_1 is a conserved MADS-Box motif found in all Class B MADS-box genes. Motif_2 occurs only in the AP1 subfamily, motif_10 only in the GGM13 subfamily, and motif_6 only in MADS-box *factor* transcription factor 2. According to these results, *WM25*, *OsMAD29*, *TaAGL14*, and *OsMAD32* have the same conserved domains and motif compositions, and thus they might share the same functionality. The amino acid sequence alignment showed that the similarity was more than 92% (Additional file 5: Figure S1, and Additional file 6: Figure S2).

### Possible molecular basis for energy deficiency model in C303A

**Fig. 9:**
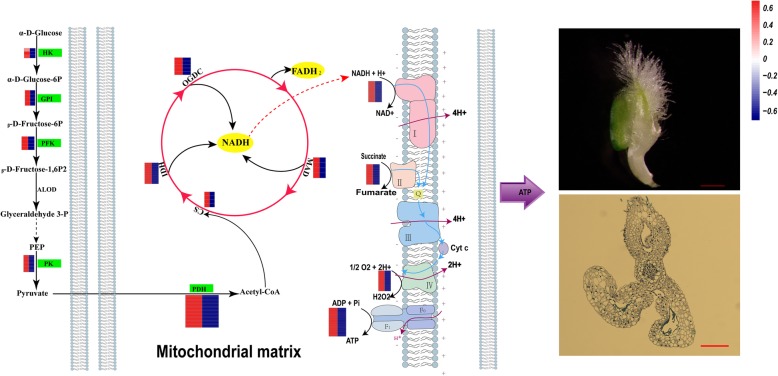
Possible gene network responsible for controlling the absence of microspores in wheat. HK: hexokinase; GPI: glucose-6-phosphate isomerase; PFK: 6-phosphofructokinase1; ALDO: fructose-bisphosphate aldolase; PK: pyruvate kinase; PDH: pyruvate dehydrogenase; CS: citrate synthase; IDH, isocitrate dehydrogenase; OGDC: 2-oxoglutarate dehydrogenase, MAD: malate dehydrogenase; I: NADH dehydrogenase; II: succinate dehydrogenase; IV: cytochrome c oxidase. V: ATP synthase. In the heat map, the left-hand side represents 303B and the right-hand side represents C303A

According to the results given above, we identified three metabolic pathways involved in carbohydrate metabolism and energy metabolism (glycolysis, TCA cycle, and pyruvate metabolism) that regulate pollen development. These results and previous studies suggest a regulatory network that might account for the lack of microspores in C303A (Fig. 9). During anther and pollen development, the amount of pyruvate as the final product of glycolysis might be decreased due to the downregulated expression of enzymes involved in glycolysis, thereby affecting the TCA cycle to some extent. In addition, the downregulated expression of genes encoding enzymes associated with the TCA cycle may contribute to reductions in a number of coenzymes (FADH_2_ and NADH) and decrease the amount of coenzymes entering the electron transport chain. The downregulation of antioxidant enzymes and key complexes represses electron transfer in the electron transport chain and the electrons then transfer directly to molecular oxygen to produce excessive amounts of reactive oxygen species (ROS). Upregulation of the activities of antioxidant enzymes disrupts the balance of the antioxidant defense system. ROS cannot be eliminated immediately and this increases the consumption of ATP, so the production of H_2_O_2_ might finally lead to the lack of microspores in C303A.

### Validation of key DEGs by real-time quantitative PCR (RT-qPCR)

**Fig. 10:**
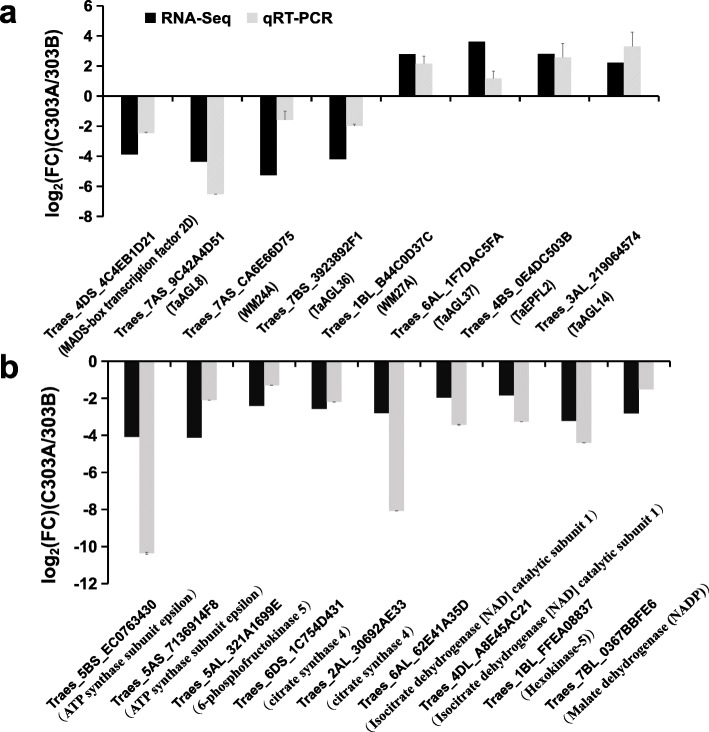
RT-qPCR validation of the RNA sequencing results for some differentially expressed genes (DEGs). Log2 (FC) represents the logarithm of the fold change in expression for C303A relative to 303B. DEGS related to pistillody (**a**) and DEGs involved in the energy metabolism process (**b**)

The transcriptional expression levels of 17 key DEGs involved in energy metabolism and pistillody were analyzed by RT-qPCR to validate the sequencing data and possible pathways (Fig. 10). Quantitative differences were found between the two methods but the trends were the same. The discrepancies in the gene expression levels were reasonable because of the different methods employed. Overall, the results demonstrated that our sequencing results were accurate and reliable, thereby confirming the possible pathways related to stamen and pollen development.

### Analysis of DEGs in energy metabolism pathway, and determination of ATP content and activities of related enzymes

**Fig. 11:**
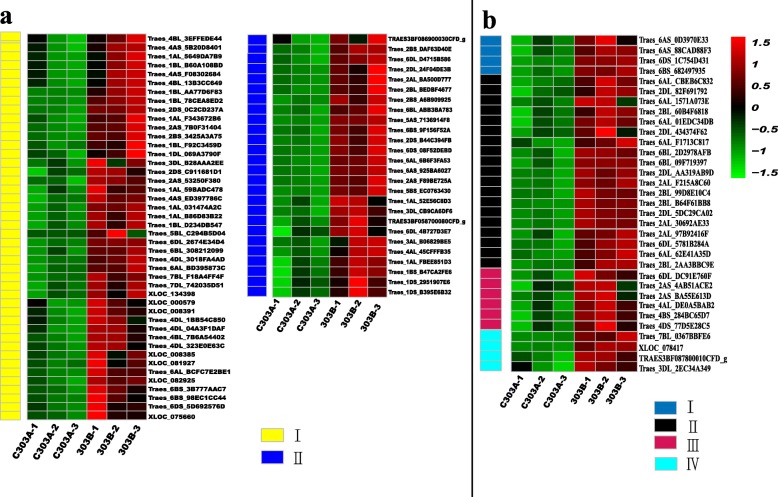
Hierarchical clustering analysis of differentially expressed genes (DEGs) in the energy metabolism pathway during the binucleate stage. **a** DEGs involved in electron transport chain, including NADH dehydrogenase (a–I) and ATP synthase (a–II). **b** DEGS involved in citric acid cycle, including citrate synthase (b-I), isocitrate dehydrogenase (B-II), 2-oxoglutarate dehydrogenase (b-III), and malate dehydrogenase (b-IV)

**Fig. 12:**
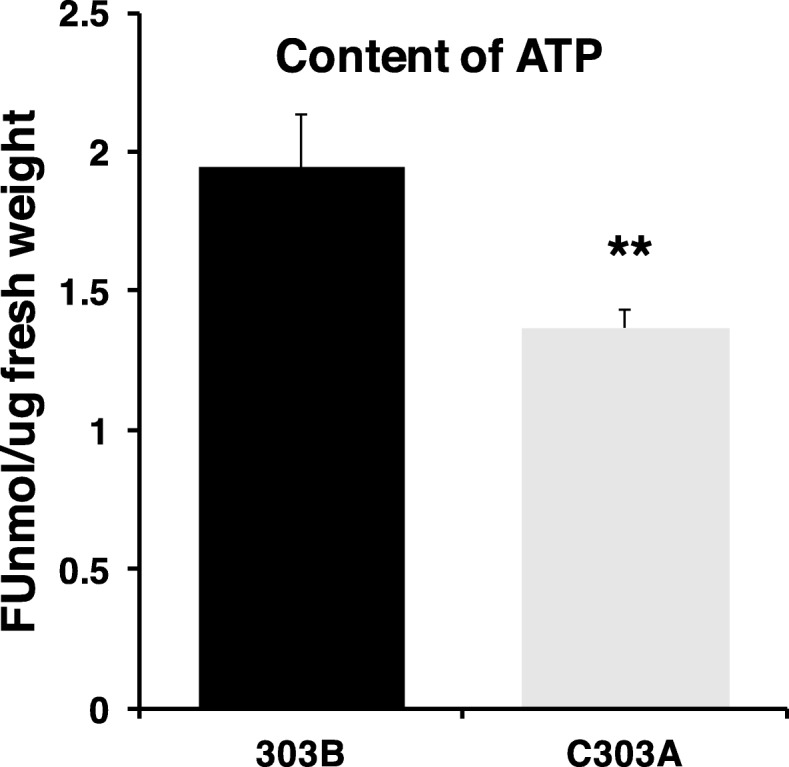
ATP contents of stamens from the maintainer line 303B and sterile line C303A during the binucleate stage. Data represent the mean and standard deviation based on three replicates. ** *p < 0.01*

The DEGs were annotated and analyzed using the KEGG pathway database to identify the DEGs related to energy metabolism. In total, 82 DEGs were annotated in the energy synthesis pathway, including NADH dehydrogenase, ATP synthase, citrate synthase, isocitrate dehydrogenase, 2-oxoglutarate dehydrogenase, and malate dehydrogenase. A heat map was prepared to analyze the expression levels of DEGs in the BNS (Fig. 11), and the results showed that the expression levels of energy synthesis pathway genes were significantly downregulated in the BNS. We measured the amount of ATP in the BNS to further confirm the accuracy of the results presented above (Fig. 12). The abundances of genes encoding enzymes related to energy metabolism were significantly lower in C303A than 303B, as shown by the heat map in Fig. 8. Due to the changes in energy metabolism-related enzymes in the pistillody stamens of C303A, we consider that the ATP level was lower in the pistillody stamens compared with the 303B stamens. The assays showed that the ATP content was also significantly lower in the C303A stamens than the 303B stamens. Thus, we hypothesize that the genes involved in energy metabolism are associated with pistillody in C303A.

### ROS assay and activities of antioxidant enzymes

**Fig. 13:**
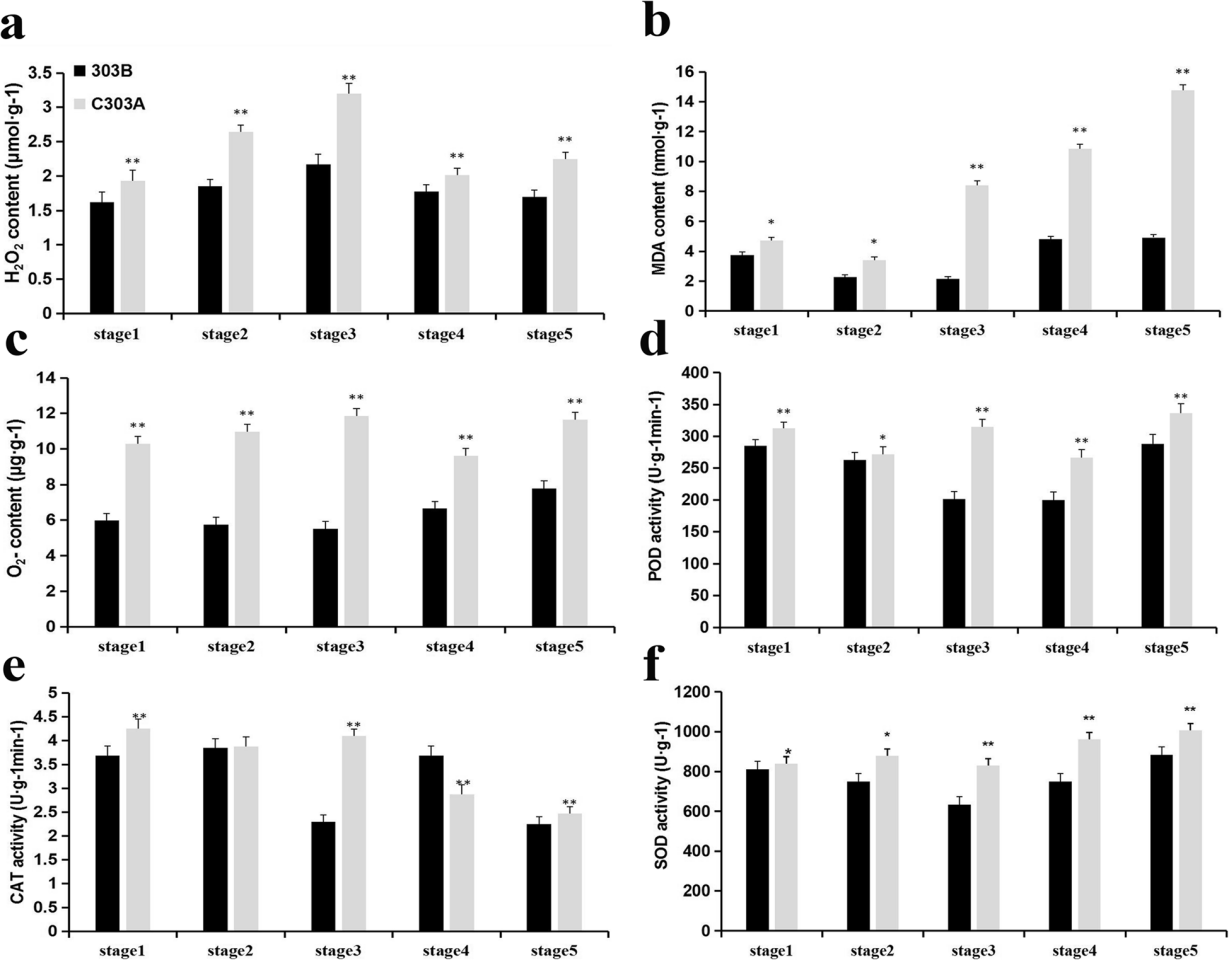
H_2_O_2_ production rate (**a**), MDA (**b**) and O^2−^ (**c**) contents, and activities of peroxidase (POD) (**d**), catalase (CAT) (**e**), and superoxide dismutase (SOD) (**f**) in developing stamens. Stage1 (Tetrad stage, TDS), stage2 (Early uninucleate stage, EUNS), Stage3 (Later uninucleate stage, LUNS), Stage4 (Binucleate stage, BNS), Stage5 (trinucleate stage, TNS). Data represent the mean and standard deviation based on three replicates. (** *p < 0.01*, * *p < 0.05*)

Compared with fertile wheat, physiological studies have shown that sterile wheat accumulates more H_2_O_2_ and malondialdehyde (MDA), and the O^2−^ generation rate is higher [16, 17]. Thus, we determined the O^2−^ generation rate as well as the H_2_O_2_ and MDA contents during all of the anther developmental stages (Fig. 13). Moreover, in order to directly determine the O^2−^ or H_2_O_2_ contents, we stained the normal and pistillody anthers using nitroblue tetrazolium (NBT) and 3,3′-diaminobenzidine (DAB) (Additional file 7: Figure S3, and Additional file 8: Figure S4). The ROS production rate was significantly higher in C303A than its maintainer line during all of the anther development stages. In addition, the O^2−^, H_2_O_2_, and MAD contents were elevated continuously in C303A, with the peak values in the uninucleate stage, whereas the MDA contents peaked in the TNS. Therefore, these results suggest that the excessive accumulation of ROS may have led to the abnormal development of tapetal cell in the C303A stamens. Furthermore, we measured the activities of antioxidant enzymes comprising catalase (CAT), superoxide dismutase (SOD), and peroxidase (POD). The SOD and POD activities remained high throughout the pollen development process in C303A, whereas the CAT activity was only high in the pistillody stamens from the tetrad stage to the later uninucleate stage. These findings suggest that the upregulated activities of antioxidant enzyme reflected the extreme accumulation of ROS in C303A, which disrupted the balance of the antioxidant defense system to cause pistillody in C303A.

## Discussion

Wheat is an allohexaploid with three genomes (A, B, and D) comprising approximately 17 Gb. The development of common wheat involved three primitive ancestral species and two natural hybrids. *Triticum urartu*, *Aegilops speltoides*, and *Aegilops tauschii* were the progenitor species for the wheat genome. Compared with rice, corn, and other crops, basic molecular research into wheat is still relatively weak and the genetic background is relatively poor. In recent years, RNA-Seq based on Illumina sequencing platform has been implemented as a reliable tool with a wide range of applications, including biological processes at the cell type-specific level, to address fundamental questions related to plant biology on an evolutionary time scale.

In this study, we performed comprehensive RNA-seq analyses of the stamens from C303A and 303B in the BNS. Compared with 303B, we detected many DEGs involved in carbohydrate and energy metabolism processes in the stamens of C303A where large numbers of these DEGs decreased in the BNS. We suggest that the decreased or abated expression of these DEGs was related to pistillody. The reduced expression of these DEGS may have led to an insufficient cellular energy supply and abnormal starch synthesis, thereby disrupting the material and energy metabolism balance in stamens and the failure to produce pollen grains.

### Starch and sucrose metabolism affected stamen development

The natural development of microspores demands a supply of carbohydrates, including starch, and amino acids to form proteins [18]. The polysaccharide contents are significantly higher during the conversion from sterility to fertility in the YS type thermo-sensitive male sterile wheat line A3314 [19]. Starch is the most common carbohydrate storage form in cells and the main source of energy for metabolism in plants. A previous study of the abortion of nuclear male sterile anthers in cotton showed that less starch accumulated in the male sterile line, and thus anther abortion was attributed to disrupted soluble sugar transport or starch synthesis [20]. The growth and development of wheat stamens are closely associated with carbohydrate metabolism because carbohydrates provide the energy required for the development of the photosynthetic apparatus in wheat. Sucrose and starch are the main forms of carbohydrate and their metabolism is crucial for plants, where this complex physiological process involves dozens of enzymes. The rate-limiting enzyme in starch synthesis is AGPase, which reversibly catalyzes the substrate adenosine diphosphate glucose during the synthesis of starch. In monocots, AGPase comprises two large subunits and two small subunits, which interact and polymerize into the native heterotetrameric enzyme structure [21, 22]. The small subunit is the catalytic center of the enzyme and the key site for enzyme allosteric effects [23]. The two subunits are encoded by different genes and studies of *Arabidopsis* have shown that they are functionally interdependent. In the present study, we found that the AGPase level was decreased during the BNS in C303A. Moreover, exopolygalacturonase and alpha-glucan phosphorylase are involved in the synthesis and accumulation of starch, where they activate the corresponding metabolic pathways to enhance starch accumulation and synthesis. Thus, these results demonstrate that starch and sucrose metabolism was probably weaker in the C303A anthers than the 303B anthers, which was closely related to the lack of pollen in C303A.

### DEGs involved in energy metabolism

The normal energy metabolism processes in plants can satisfy their growth and development requirements. Energy metabolism mainly includes major processes such as photosynthesis, glycolysis, oxidative phosphorylation, and the TCA cycle. Energy metabolism processes require the involvement of many related genes or proteins. Numerous studies have shown that the abnormal expression of these genes in the stamen will inevitably interfere with the energy supply for pollen development. For example, the fluorescein-luciferase method was used to determine the ATP contents of the anthers, where the results showed that the ATP contents were much lower in normal anthers from the early stage of pollen grain development to the maturation phase [24]. Similarly, Xia and Liu [25] used the same method to determine the ATP contents of stamens in different development stages by using a corn CMS line and its maintainer system. The results showed that the ATP contents in the anther were significantly lower in the sterile line than the maintainer, and it was suggested that a large amount of energy is consumed during the pollen abortion process because the formation of microspores requires large amounts of energy and nutrients. In the present study, the absence of microspores in C303A may have been due to the downregulation of energy metabolism genes during the formation of pistillody stamens. Moreover, hexokinase-5, 6-phosphofructokinase 5, and pyruvate kinase play vital roles in the glycolysis pathway, and their expression levels were significantly downregulated in C303A. These genes may affect stamen development. In particular, significant downregulation of these genes directly leads to decreases in the levels of many respiratory substrates as well as interfering with the electron transmit chain (ETC) in mitochondria. Moreover, the expression levels of many DEGs related to ETC and TCA decreased in this stage, and our assays of the ATP contents supported the possible involvements of this mechanism (Fig. 12). Thus, we suggest that the energy levels were lower in the pistillody stamens of C303A than the 303B stamens, and they could not meet the basic energy requirements for microspores in C303A so the pistillody stamens did not form microspores.

### DEGs related to pistillody

When a stamen undergoes a homeotic transformation into a pistil-like structure, the phenomenon is called pistillody, and it can cause stable and complete male sterility, as found in species such as *Arabidopsis* [26] and *Antirrhinum* [14, 27]. In the present study, pistillody occurred in the CMS wheat line C303A with *Ae. crassa* cytoplasm, but the pistillody phenomenon was not clear, so we used RNA-seq to elucidate the molecular mechanism responsible for pistillody by determining the genes that affected flower development.

PI and AP3 proteins bind together specifically to function as class-B proteins that localize to the nucleus [28]. Moreover, according to the ABC model, class-B MADS-box genes are expressed in both whorl 3 and whorl 2 during the development of stamens and lodicules in *Arabidopsis* [26]. A previous study showed that pistillody is due to deficient *WPI1* gene expression in whorl 3 [11]. Furthermore, in monocots, a maize class-B gene-deficient mutant called silky1 exhibits male sterility because of the homeotic conversion of stamens into carpels [29]. Moreover, transgenic rice that express antisense RNA for the class-B gene *OsMADS4* possess stamens that are modified into a carpel-like organ [30]. Similarly, pistillody is caused by changes in the expression patterns of class B MADS-box genes in wheat. In the present study, we identified a paralogous gene (*TaAGL14*) with a similar role to *OsMADS32*. In a previous study of cfo1–1, it was shown that strong *OsMADS32* alleles increased the number of pistils and *OsMADS32* was required for pistil development and floral meristem determinacy. Furthermore, we found that the *TaAGL14* gene was upregulated in C303A, and thus class-B MADS-box genes may contribute to pistil development in wheat.

MIKC-type MADS-box transcription factor genes are related to various critical developmental processes such as ovule development, vegetative growth, flower morphogenesis, and fruit formation [31, 32]. The expression pattern of MADS-box transcription factor *WM27A* is compatible with its function as a class D gene, where it regulates ovule identity specification according to the “ABCDE” model of flower development [33]. A previous study found that *WM27A* was strongly expressed in the pistils and caryopses, but with weak expression in the stamens and extremely high expression during late spike development. Moreover, the phylogenetic tree constructed based on the nucleotide sequences of a MADS-box transcription factor *TaMS-MADSbox* (GenBank accession number: 36925702) linked to fertility conversion in male sterile wheat lines [34] as well as the MADS-box transcription factors identified using our sequencing data showed that *WM27A* and *TaMS-MADSbox* are on the same branch. In the present study, the expression level of *WM27A* was upregulated in C303A, which is similar to the results obtained in previous studies. The EPIDERMAL PATTERNING FACTOR-LIKE (EPFL) family has various functions in plant growth and development, such as guiding the inflorescence architecture and pedicel length. The *TaEPFL1* gene encodes a secreted peptide with an essential role in stamen development in wheat [35]. Moreover, the *TaEPFL1* gene is expressed at an abnormally high level in pistillody stamens compared with pistils and stamens. We found that genes encoding EPFL proteins in C303A have possible roles in pistillody. However, the mechanisms that allow these genes to control stamen and pistil development in wheat require further investigation.

## Conclusions

The pistil developed normally in the CMS wheat line C303A with *Ae. crassa* cytoplasm but the stamens were ultimately aborted and they released no pollen when mature. According to paraffin section analyses, the stamens began to transform into pistils or pistil-like structures during the BNS. RNA-Seq showed that the downregulated expression of genes involved in carbohydrate and energy metabolism was closely related to pollen development. Thus, we suggested a possible regulatory network where the downregulation of key genes in this network is mainly responsible for the lack of microspores in C303A. In addition, assays of ATP, ROS, and ROS scavenging enzymes supported the existence of the proposed regulatory network. Furthermore, the regulation of pollen and stamen development is affected by members of the MADS-box transcription factor family. Thus, the conversion of stamens into pistil-like structures in C303A is due to the effects of multiple genes rather than a single gene. Our findings may facilitate further mechanistic studies of stamen and pollen development in CMS wheat.

## Methods

### Plant materials, plant growth, and anther collection

CMS line C303A and its isomaintainer line 303B were employed in this study. All of the experimental materials were cultivated using conventional methods at the experimental station of Northwest A&F University, Yangling, China. The pollen development stages comprising the tetrad stage, early uninucleate stage, later uninucleate stage, BNS, and TNS were identified using acetic red dyeing methods. C303A lacks microspores so we determined the developmental stages in C303A based on comparisons with the characteristics of 303B. Previous studies have shown that the spike development stages are the same in 303B and C303A [36, 37], so we determined the average spike length and external morphological characteristics during each period in 303B. In the tetrad stage, the spike length was 3.7 cm in 303B and the apical spike was in the middle of the second top leaves and the third top leaves. In the early uninucleate stage, the spike length was 10.5 cm in 303B and the apical spike was in the middle of the second top leaves and the flag leaf. In the later uninucleate stage, the spike length was 12.9 cm in 303B and the awn was fully grown. In the BNS, the spike length was 11.4 cm in 303B and the wheat ear was about two-third complete. In the TNS, the spike length was 14.5 cm in 303B and the whole spike was opened out, while some glumes were open with the anthers exposed. According to these characteristics of 303B, we collected flowers and stamens in different developmental stages to obtain morphological and cytological observations during heading and anthesis. Moreover during the BNS, the stamens were collected from three individual fertile plants (303B-1, 303B-2, and 303B-3) and three individual sterile plants (C303A-1, C303A-2, and C303A-3) for transcriptome sequencing with three biological replicates. The harvested stamens were quickly flash frozen in liquid nitrogen and then stored at − 80 °C for further analysis.

### Morphological analysis

Stamens and florets were carefully removed with tweezers and anatomical needles. The morphological characteristics of the florets and stamens were observed and photographed under a microscope (Preiser Scientific, Louisville, KY, USA). Mature pollen grains were dipped in a drop of I_2_–KI solution. All of the samples were placed in formalin–acetic acid–alcohol fixative solution and stored in a refrigerator at 4 °C. The specimens were then dehydrated using a graded ethanol series, infiltrated with xylene, and embedded in paraffin. Sections measuring 5 μm were placed onto gelatine-coated glass slides (Sigma-Aldrich) and stained with toluidine blue [38]. The stamens in different stages were observed using a DS-U2 high-resolution camera mounted on a Nikon ECLIPSE E600 microscope (Nikon, Tokyo, Japan).

In order to directly determine the O^2−^ or H_2_O_2_ contents, normal and pistillody anthers were stained using NBT and DAB. The anthers were vacuum infiltrated with 0.1% DAB solution (pH 6.5) for 24 h and after standing for 30 min in the light, the samples were cleared by boiling in alcohol:lactophenol (2:1) for 5 min and rinsing twice with 50% ethanol. To detect O^2−^, the samples were vacuum infiltrated (25 psi for 5 min) with 0.05 M phosphate-buffered saline (pH 7.4) containing 0.5 mM NBT. The samples were incubated at room temperature for 1 h and the reaction was stopped with 95% ethanol. Chlorophyll was removed by repeated treatment with ethanol. The stained samples were placed on a slide and observed under a microscope.

### Total RNA extraction, cDNA library preparation, and Illumina sequencing

Total RNA was extracted from C303A and 303B stamens during the BNS stage using TRIzol reagent (Takara Biotechnology, Dalian, China). The integrity of the total RNA was confirmed by 1% agarose gel electrophoresis, where OD260 and OD280 were detected using a Nanodrop spectrophotometer for the samples, before determining the RNA purity, sample concentration, RIN (The RNA Integrity Number) value, and 28S/18S value with an Agilent 2100 Bioanalyzer (Agilent Technologies, Santa Clara, CA, USA).

All of the samples were submitted to Sagene Biotech Co. Ltd. (Guangzhou, China) for library generation and sequencing, and the eukaryotic mRNA was enriched with Oligo (dT) beads. Fragmentation buffer was used to break the mRNA into short fragments. cDNA was synthesized using mRNA as the template with random primers. DNA polymerase I, RNase H, dNTP, and buffer were added to synthesize second-strand DNA, before purifying with a QiaQuick PCR Extraction Kit (GeneStar Co. Ltd., Beijing, China). The purified cDNA was end repaired and poly (A) was added. Finally, PCR amplification was performed and the PCR product was purified with AMPure XP beads (Beckman Coulter, Brea, CA, USA) to obtain the final library. After quality checking the library, different libraries were pooled according to the requirements in terms of the effective concentration and target data volume, before Illumina HiSeq sequencing.

### Raw data filtering and transcript splicing

The raw data filtering process was conducted as follows: (1) removal of reads with adapter sequences; (2) elimination of reads containing more than 10% unknown nucleotides (N); and (3) excluding low-quality reads. In order to ensure the quality of the information obtained, raw reads were filtered to yield clean reads and subsequent analyses were based on the clean reads.

Chinese Spring wheat was used as the reference genome (Ensembl release 31 IWGSC1.0 + NC_002762.1). Clean reads were compared with reference genome sequences using HISAT2 (v2.2.0.4, https://ccb.jhu.edu/software/hisat2/index.shtml) [39] and the alignment of transcripts was assembled with Cufflinks [40] (v2.1.1, (http://cole-trapnell-lab.github.io/cufflinks/).

### Screening and analysis of DEGs

The input data for DEG analysis comprised the read count data obtained by gene expression level analysis. First, the read count data were standardized based on the trimmed mean of M values (TMM) [16]. These genes were then used for differential expression analysis. The thresholds comprised q-value < 0.005 and |log 2 (fold change) | > 1. For DEGs, if log 2(fold change) > 0, the DEG was considered to be upregulated; otherwise, it was considered to be downregulated [41].

### GO function annotation, classification, and KEGG analysis

GO enrichment analysis was conducted for all of the DEGs using the GO database. First, mapping was performed by calculating the number of genes per term and significantly enriched DEGs were then compared with the entire genome background [42].

KEGG is the main free database for pathway analysis. Significantly enriched KEGG pathways were determined by conducting a hypergeometric test for the significantly enriched DEGs relative to all of the annotated genes. KOBAS (2.0) (http://kobas.cbi.pku.edu.cn) was employed and the FDR parameter was subjected to Benjamini–Hochberg correction [43] for pathway enrichment analysis. A pathway with FDR ≤ 0.05 was designated as a pathway significantly enriched with DEGs [44].

### Physiological indexes

The ATP content was determined spectrophotometrically according to the protocol supplied with an ATP Kit (Comin Biotechnology Co. Ltd., Suzhou, China). Three ground C303A anther samples (0.1 g each) were placed in a centrifuge tube and 1 mL of acid extract was added, before stirring well. The mixture was homogenized for 10 min, centrifuged at 8000 r/min and 4 °C for 10 min, before placing the supernatant in another centrifuge tube, adding 500 μL of chloroform and mixing well by shaking. This mixture was centrifuged at 10000 r/min and 4 °C for 3 min, and the supernatant was placed on ice for ATP content analysis using a spectrophotometric method. The ATP contents were measured in 303B anthers using the same method.

The H_2_O_2_, O^2−^, and MDA contents, and CAT, POD, and SOD activity levels were determined as described previously [45, 46].

### Phylogenetic tree construction and analysis of characteristic conserved domain structures and motif compositions

To identify all of the candidate MADS-box genes based on our RNA-seq results, systematic BLASTp (https://blast.ncbi.nlm.nih.gov/Blast.cgi) searches were conducted against the wheat reference genome (https://plants.ensembl.org/Triticum_aestivum/Info/Index) and the NCBI database, where the screening criteria were E < 1e-10 and protein length > 200 amino acids. The full-length amino acid sequences of the MADS-box genes in maize (*Zea mays*), *Arabidopsis* (*Arabidopsis thaliana*), and rice (*Oryza sativa*) were obtained from an online database (https://www.ncbi.nlm.nih.gov/) using NCBI BLASTp tools. The maximum likelihood method [47] was then used to reconstruct the phylogenetic tree with MEGA6 (Molecular Evolutionary Genetics Analysis Version 6.0).

We submitted all of the class B MADS-box protein sequences to the MEME Suite web server (http://meme-suite.org/) for motif composition analysis. Conserved domains were obtained from the NCBI website (https://www.ncbi.nlm.nih.gov/Structure/bwrpsb/bwrpsb.cgi).

### RT-qPCR analysis

Fifteen DEGs correlated with fertility conversion were randomly selected and Primer-NCBI (https://www.ncbi.nlm.nih.gov/tools/primer-blast/) was used to design the primers (Additional file 3: Table S3) for RT-qPCR analysis. RNA was reverse transcribed into cDNA using a first strand cDNA synthesis kit (GeneStar Co. Ltd., Beijing, China), where cDNA was used as the template and the *Actin* gene as an internal standard. A TaKaRa (Japan) SYBR® Premix Ex TaqTM II (Tli RNaseH Plus) kit was used. Quantitative fluorescence detection was performed with the QuantStudio® 7 Flex Real-Time PCR system (Applied Biosystems, Shanghai, China). Three technical replicates were performed for each sample and the relative expression levels were determined according to the 2^−ΔΔCt^ method [48].

## Supplementary information

**Additional file 1: Table S1.** B706-vs-C706A GO enrichment in biological process.

**Additional file 2: Table S2.** B706-vs-C706A pathway enrichment.

**Additional file 3: Table S3.** Phylogenetic tree based on MADS-box transcription factor genes in different plant species.

**Additional file 4: Table S4.** Sequence-specific primers used for RT-qPCR.

**Additional file 5: Figure S1.** Amino acid sequence alignments for *TaAGl14* and *OsMADS32*.

**Additional file 6: Figure S2.** Amino acid sequence alignments for *WM25* and *OsMADS29*.

**Additional file 7: Figure S3:** Stamens of plants stained with DAB to monitor H_2_O_2_ accumulation after 24 h in 303B (a-e) and C303A (f-j). Scale bars represent 200 μm.

**Additional file 8: Figure S4:** Stamens of plants stained with NBT to monitor O^2−^ accumulation after 1 h in 303B (a-e) and C303A (f-j). Scale bars represent 200 μm.

## Data Availability

All data sets supporting the conclusions of this study are included within the article (and additional files). The dataset generated during the current study including the RNA-Seq data are available at NCBI Sequence Read Archive (SRA) database with BioProject ID # PRJNA596597.

## Abbreviations

BNS: Binucleate stage
CMS: Cytoplasmic male sterility
DAB: 3,3′-Diaminobenzidine
DEGs: Differentially expressed genes
GO: Gene Ontology
KEGG: Kyoto Encyclopedia of Genes and Genomes
NBT: Nitroblue tetrazolium
RNA-Seq: RNA sequencing technology
TNS: Trinucleate stage

## References

[1] Paux E, Sourdille P, Salse J, Saintenac C, Choulet F, Leroy P, Korol A, Michalak M, Kianian S, Spielmeyer W (2008). A physical map of the 1-gigabase bread wheat chromosome 3B. Science.

[2] Singh RP, Singh PK, Rutkoski J, Hodson DP, He X, Jorgensen LN, Hovmoller MS, Huerta-Espino J (2016). Disease impact on wheat yield potential and prospects of genetic control. Annu Rev Phytopathol.

[3] Gao FM, Ma DY, Yin GH, Rasheed A, Dong Y, Xiao YG, Xia XC, Wu XX, He ZH (2017). Genetic progress in grain yield and physiological traits in Chinese wheat cultivars of southern yellow and Huai Valley since 1950. Crop Sci.

[4] Zhang GM, Ye JL, Jia YL, Zhang LL, Song XY (2018). iTRAQ-based proteomics analyses of sterile/fertile anthers from a thermo-sensitive cytoplasmic male-sterile wheat with *Aegilops kotschyi* cytoplasm. Int J Mol Sci.

[5] Chen L, Liu YG (2014). Male sterility and fertility restoration in crops. Annu Rev Plant Biol.

[6] Murai K, Tsunewaki K (1993). Photoperiod-sensitive cytoplasmic male sterility in wheat with *Aegilops crassa* cytoplasm. Euphytica.

[7] Zhu Y, Saraike T, Yamamoto Y, Hagita H, Takumi S, Murai K (2008). *orf260cra,* a novel mitochondrial gene, is associated with the homeotic transformation of stamens into pistil-like structures (pistillody) in alloplasmic wheat. Plant Cell Physiol.

[8] Coen ES, Meyerowitz EM (1991). The war of the whorls: genetic interactions controlling flower development. Nature.

[9] Pelaz S, Ditta GS, Baumann E, Wisman E, Yanofsky MF (2000). B and C floral organ identity functions require *SEPALLATA* MADS-box genes. Nature.

[10] Theissen G (2001). Development of floral organ identity: stories from the MADS house. Curr Opin Plant Biol.

[11] Hama E, Takumi S, Ogihara Y, Murai K (2004). Pistillody is caused by alterations to the class-B MADS-box gene expression pattern in alloplasmic wheats. Planta.

[12] Murai K, Takumi S, Koga H, Ogihara Y (2002). Pistillody, homeotic transformation of stamens into pistil-like structures, caused by nuclear-cytoplasm interaction in wheat. Plant J.

[13] Goto K, Meyerowitz EM (1994). Function and regulation of the *Arabidopsis* floral homeotic gene *PISTILLATA*. Genes Dev.

[14] Trobner W, Ramirez L, Motte P, Hue I, Huijser P, Lonnig WE, Saedler H, Sommer H, Schwarz-Sommer Z (1992). GLOBOSA: a homeotic gene which interacts with *DEFICIENS* in the control of *Antirrhinum* floral organogenesis. EMBO J.

[15] Ye J, Fang L, Zheng H, Zhang Y, Chen J, Zhang Z, Wang J, Li S, Li R, Bolund L (2006). WEGO: a web tool for plotting GO annotations. Nucleic Acids Res.

[16] Li J, Ding X, Han S, He T, Zhang H, Yang L, Yang S, Gai J (2016). Differential proteomics analysis to identify proteins and pathways associated with male sterility of soybean using iTRAQ-based strategy. J Proteome.

[17] Liu ZH, Shi XY, Li S, Zhang LL, Song XY (2018). Oxidative stress and aberrant programmed cell death are associated with pollen abortion in isonuclear alloplasmic male-sterile wheat. Front Plant Sci.

[18] Geng XX, Ye JL, Yang XT, Li S, Zhang LL, Song XY (2018). Identification of proteins involved in carbohydrate metabolism and energy metabolism pathways and their regulation of cytoplasmic male sterility in wheat. Int J Mol Sci.

[19] Song XY, Hu YG, Ma LJ, Li HB, He BR (2009). Changes of material content in panicles and leaves of YS type thermo-sensitive male sterile wheat line A3314 during transfer from sterility to fertility. J Northwest A F Univ.

[20] Song XL, Sun XZ, Wang HG (2004). Biochemical changes in anthers of “Dong a” genetic male sterile lines of cotton. Acta Botan Boreali-Occiden Sin.

[21] Tang XJ, Peng C, Zhang J, Cai Y, You XM, Kong F, Yan HG, Wang GX, Wang L, Jin J (2016). ADP-glucose pyrophosphorylase large subunit 2 is essential for storage substance accumulation and subunit interactions in rice endosperm. Plant Sci.

[22] Kang GZ, Wang YH, Liu C, Shen BQ, Zheng BB, Feng W, Guo TC (2010). Difference in AGPase subunits could be associated with starch accumulation in grains between two wheat cultivars. Plant Growth Regul.

[23] Cheng C, Hu J, Zhi Y, Su JJ, Zhang XK, Huang BQ (2015). Cloning and characterization of ADP-glucose pyrophosphorylase small subunit gene in *Cyperus esculentus* (yellow nutsedge). Genet Mol Res.

[24] Li J, Pandeya D, Jo YD, Liu WY, Kang BC (2013). Reduced activity of ATP synthase in mitochondria causes cytoplasmic male sterility in chili pepper. Planta.

[25] Tao X, Jilin L (1994). Cytochrome oxidase activity and ATP content of male-sterile cytoplasm in maize (*Zea mays* L.). J North China Agric.

[26] Jack T, Brockman LL, Meyerowitz EM (1992). The homeotic gene *APETALA3* of *Arabidopsis thaliana* encodes a MADS box and is expressed in petals and stamens. Cell.

[27] Sommer H, Beltran JP, Huijser P, Pape H, Lonnig WE, Saedler H, Schwarz-Sommer Z (1990). Deficiens, a homeotic gene involved in the control of flower morphogenesis in *Antirrhinum majus*: the protein shows homology to transcription factors. EMBO J.

[28] McGonigle B, Bouhidel K, Irish VF (1996). Nuclear localization of the *Arabidopsis APETALA3* and PISTILLATA homeotic gene products depends on their simultaneous expression. Genes Dev.

[29] Mizzotti C, Mendes MA, Caporali E, Schnittger A, Kater MM, Battaglia R, Colombo L (2012). The MADS box genes *SEEDSTICK* and *ARABIDOPSIS* Bsister play a maternal role in fertilization and seed development. Plant J.

[30] Kang HG, Jeon JS, Lee S, An G (1998). Identification of class B and class C floral organ identity genes from rice plants. Plant Mol Biol.

[31] Becker A, Theissen G (2003). The major clades of MADS-box genes and their role in the development and evolution of flowering plants. Mol Phylogenet Evol.

[32] Paolacci AR, Tanzarella OA, Porceddu E, Varotto S, Ciaffi M (2007). Molecular and phylogenetic analysis of MADS-box genes of MIKC type and chromosome location of SEP-like genes in wheat (*Triticum aestivum* L.). Mol Gen Genomics.

[33] Pinyopich A, Ditta GS, Savidge B, Liljegren SJ, Baumann E, Wisman E, Yanofsky MF (2003). Assessing the redundancy of MADS-box genes during carpel and ovule development. Nature.

[34] Zhou LL, Song GQ, Li HB, Hu YG, He BR (2008). A MADS-box transcription factor related to fertility conversion in male sterile wheat lines. Acta Agron Sin.

[35] Sun QX, Qu JP, Yu Y, Yang ZJ, Wei SH, Wu YL, Yang J, Peng ZS (2019). TaEPFL1, an EPIDERMAL PATTERNING FACTOR-LIKE (EPFL) secreted peptide gene, is required for stamen development in wheat. Genetica.

[36] Liu ZH, Shi XY, Li S, Ye JL, Meng LY, Yan PJ, Zhang LL, Song XY (2017). Tapetal programmed cell death, antioxidant response and oxidative stress in wheat anthers associated with D^2^-type cytoplasmic male-sterility. Sci Agric Sin.

[37] Yao M, Ye JL, Yang ZQ, Duan Y, Meng LY, Yan PJ, Liu ZH, Zhang LL, Song XY (2015). Abortion feature and fertility restoration of five kinds of cytoplasmic male sterile wheat lines. J Triticeae Crops.

[38] Wang SP, Zhang GS, Song QL, Zhang Y, Li Z, Guo J, Niu N, Ma SC, Wang JW (2015). Abnormal development of tapetum and microspores induced by chemical hybridization agent SQ-1 in wheat. PLoS One.

[39] Roberts A, Pimentel H, Trapnell C, Pachter L (2011). Identification of novel transcripts in annotated genomes using RNA-Seq. Bioinformatics..

[40] Zararsiz G, Cosgun E (2014). Introduction to statistical methods for microRNA analysis. Methods Mol Biol.

[41] Wang LK, Feng ZX, Wang X, Wang XW, Zhang XT (2010). DEGseq: an R package for identifying differentially expressed genes from RNA-seq data. Bioinformatics.

[42] Young MD, Wakefield MJ, Smyth GK, Oshlack A (2010). Gene ontology analysis for RNA-seq: accounting for selection bias. Genome Biol.

[43] Benjamini Y, Hochberg Y (1995). Controlling the false discovery rate - a practical and powerful approach to multiple testing. J R Stat Soc.

[44] Kanehisa M, Araki M, Goto S, Hattori M, Hirakawa M, Itoh M, Katayama T, Kawashima S, Okuda S, Tokimatsu T (2008). KEGG for linking genomes to life and the environment. Nucleic Acids Res.

[45] Jacoby RP, Millar AH, Taylor NL (2010). Wheat mitochondrial proteomes provide new links between antioxidant defense and plant salinity tolerance. J Proteome Res.

[46] Ba QS, Zhang GS, Wang JS, Che HX, Liu HZ, Niu N, Ma SC, Wang JW (2013). Relationship between metabolism of reactive oxygen species and chemically induced male sterility in wheat (*Triticum aestivum* L.). Can J Plant Sci.

[47] Tamura K, Stecher G, Peterson D, Filipski A, Kumar S (2013). MEGA6: molecular evolutionary genetics analysis version 6.0. Mol Biol Evol.

[48] Lim S, Yoon H, Ryu S, Jung J, Lee M, Kim D (2006). A comparative evaluation of radiation-induced DNA damage using real-time PCR: influence of base composition. Radiat Res.

