# Supplementary material for: Comparative transcriptome analysis indicates conversion of stamens into pistil-like structures in male sterile wheat (Triticum aestivum L.) with Aegilops crassa cytoplasm
Source: BMC Genomics. 2020 Feb 4;21:124. doi: 10.1186/s12864-020-6450-2 (PMC7001380; doi:10.1186/s12864-020-6450-2)
Supplement: Supplementary file 5 — Additional file 5: Figure S1. Amino acid sequence alignments for TaAGl14 and OsMADS32. [file 12864_2020_6450_MOESM5_ESM.pdf]

```
OsMADS32      MGRGRSEIKRIENPTQRQSTFYKRRDGLFKKARELAVLCD   40
TaAGL14       MGRGRSEIKRIDNPTQRQSTFYKRRDGLFKKARELAVLCD   40
TaAGL14_copy  MGRGRSEIKRIDNPTQRQSTFYKRRDGLFKKARELAVLCD   40
Consensus     mgrgrseikri nptqrqstfykrrdglfkkarelavlcd

OsMADS32      ADLLLLLFSASGKLYHFLSPTVPSVREFVERYEATTHTKV   80
TaAGL14       ADLLLLLFSASGKLYQYLAPTVPSVKEFVERYEASTHTKV   80
TaAGL14_copy  ADLLLLLFSASGKLYQYLAPTVPSVKEFVERYEASTHTKV   80
Consensus     adlllllfsasgkly  l ptvpsv efveryea thtkv

OsMADS32      WADIRQERRAELEKVGSMCDLLEKQLRFMTVDDGEEYTVP  120
TaAGL14       WSDIRQERRRELEKVAKMCDLLEKELRFMTVDDGERYTVP  120
TaAGL14_copy  WSDIRQERRRELEKVAKMCDLLEKELRFMTVDDGERYTVP  120
Consensus     w dirqerr elekv  mcdllek lrfmtvddge ytvp

OsMADS32      SLEALEHNLEAAMRKVRSEKDRKIGGEICYLQNIIRGRQE  160
TaAGL14       SLAALEHNLEAAMHKVRSEKDRKIGGEMSYLENMIRGKQA  160
TaAGL14_copy  SLAALEHNLEAAMHKVRSEKDRKIGGEMSYLENMIRGKQA  160
Consensus     sl alehnleaam kvrsekdrkigge  yl n irg q

OsMADS32      ERYGLCDKIAHAQTLKDVECGSTSLSNGLDLKLG.FN...  196
TaAGL14       ERYGLCDKLAHAQSLKVVEGGSTSLNNGLDLKLGRFSQSI  200
TaAGL14_copy  ERYGLCDKLAHAQSLKVVEGGSTSLNNGLDLKLGRFSQSI  200
Consensus     erygl cdk ahaq lk ve gstsl ngldlklg f

OsMADS32      ............                              196
TaAGL14       QNLVTVQVSRSQ                              212
TaAGL14_copy  QNLVTVQVSRSQ                              212
Consensus
```
