# Supplementary material for: Comparative transcriptome analysis indicates conversion of stamens into pistil-like structures in male sterile wheat (Triticum aestivum L.) with Aegilops crassa cytoplasm
Source: BMC Genomics. 2020 Feb 4;21:124. doi: 10.1186/s12864-020-6450-2 (PMC7001380; doi:10.1186/s12864-020-6450-2)
Supplement: Supplementary file 6 — Additional file 6: Figure S2. Amino acid sequence alignments for WM25 and OsMADS29. [file 12864_2020_6450_MOESM6_ESM.pdf]

```
WM25             MGRGKIEIKRIENATNRQVTFSKRRGGLLKKANELAVLCD   40
OsMADS29         MGRGKIEIKRIENATNRQVTFSKRRGGLLKKANELAVLCD   40
OsMADS29_copy    MGRGKIEIKRIENATNRQVTFSKRRGGLLKKANELAVLCD   40
Consensus        mgrgkieikrienatnrqvtfskrrgglllkkanelavlcd

WM25             ARVGVVIFSSTGRMFEYSSPASSLRDLIEQYQNATNSQFE   80
OsMADS29         ARVGVVIFSSTGKMFEYCSPTCSLRELIEHYQTVTNTHFE   80
OsMADS29_copy    ARVGVVIFSSTGKMFEYCSPTCSLRELIEHYQTVTNTHFE   80
Consensus        arvgvvifsstg mfey sp  slr lie yq  tn  fe

WM25             EINHDQQIFVEMTRMRNEMEKLDGAIRRYTGDDLSSLSLA  120
OsMADS29         EINHDQQIFVEMTRMRNEMEKLDGGIRRFTGDDLSNLTLA  120
OsMADS29_copy    EINHDQQIFVEMTRMRNEMEKLDGGIRRFTGDDLSNLTLA  120
Consensus        einhdqqifvemtrmrnemekldg irr tgddls l la

WM25             DVNDIEQQLEFSVAKVRARKHQLLNQQLDNLRRKEHILED  160
OsMADS29         DINDLEQQLEFSVTKVRARKHQLLNQQLDNLRRKEHILED  160
OsMADS29_copy    DINDLEQQLEFSVTKVRARKHQLLNQQLDNLRRKEHILED  160
Consensus        d nd eqqlefsv kvrarkhqllnqqldnlrrkehiled

WM25             QNSFLCRMISENQH......GSDRKMAVMPPVLSMLTPAF  194
OsMADS29         QNSFLCRMINENHHQAAVGGGDVKAMVEMAPVLSMLT...  197
OsMADS29_copy    QNSFLCRMINENHHQAAVGGGDVKAMVEMAPVLSMLT...  197
Consensus        qnsflcrmi en h      g    m  m pvlsmlt

WM25             PATPYYTGEESSSTALQLTSPQLQLHAAEAAGFRLQPTQP  234
OsMADS29         .AAPAYYGEESSSTALQLTPPLHAVDAAAAAGFRLQPTQP  236
OsMADS29_copy    .AAPAYYGEESSSTALQLTPPLHAVDAAAAAGFRLQPTQP  236
Consensus         a p y geessstalqlt p     aa aagfrlqptqp

WM25             NLQDPACSS.....LHAGHGLHL                   252
OsMADS29         NLQDPGCSSSSFHAAAAGHGLQL                   259
OsMADS29_copy    NLQDPGCSSSSFHAAAAGHGLQL                   259
Consensus        nlqdp css       aghgl l
```
