# Supplementary figures and images for: Comparative transcriptome analysis indicates conversion of stamens into pistil-like structures in male sterile wheat (Triticum aestivum L.) with Aegilops crassa cytoplasm
Source: BMC Genomics. 2020 Feb 4;21:124. doi: 10.1186/s12864-020-6450-2 (PMC7001380; doi:10.1186/s12864-020-6450-2)

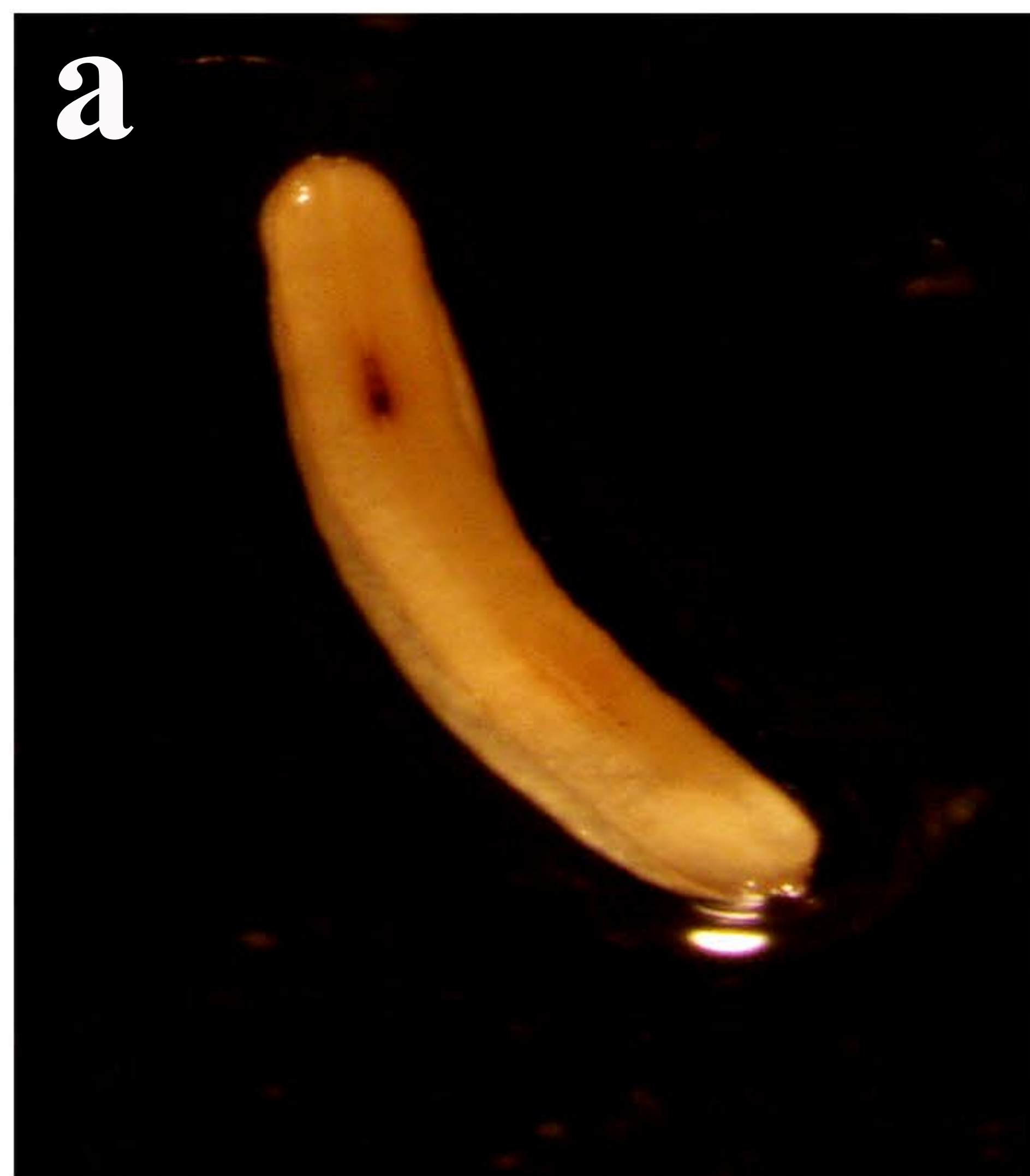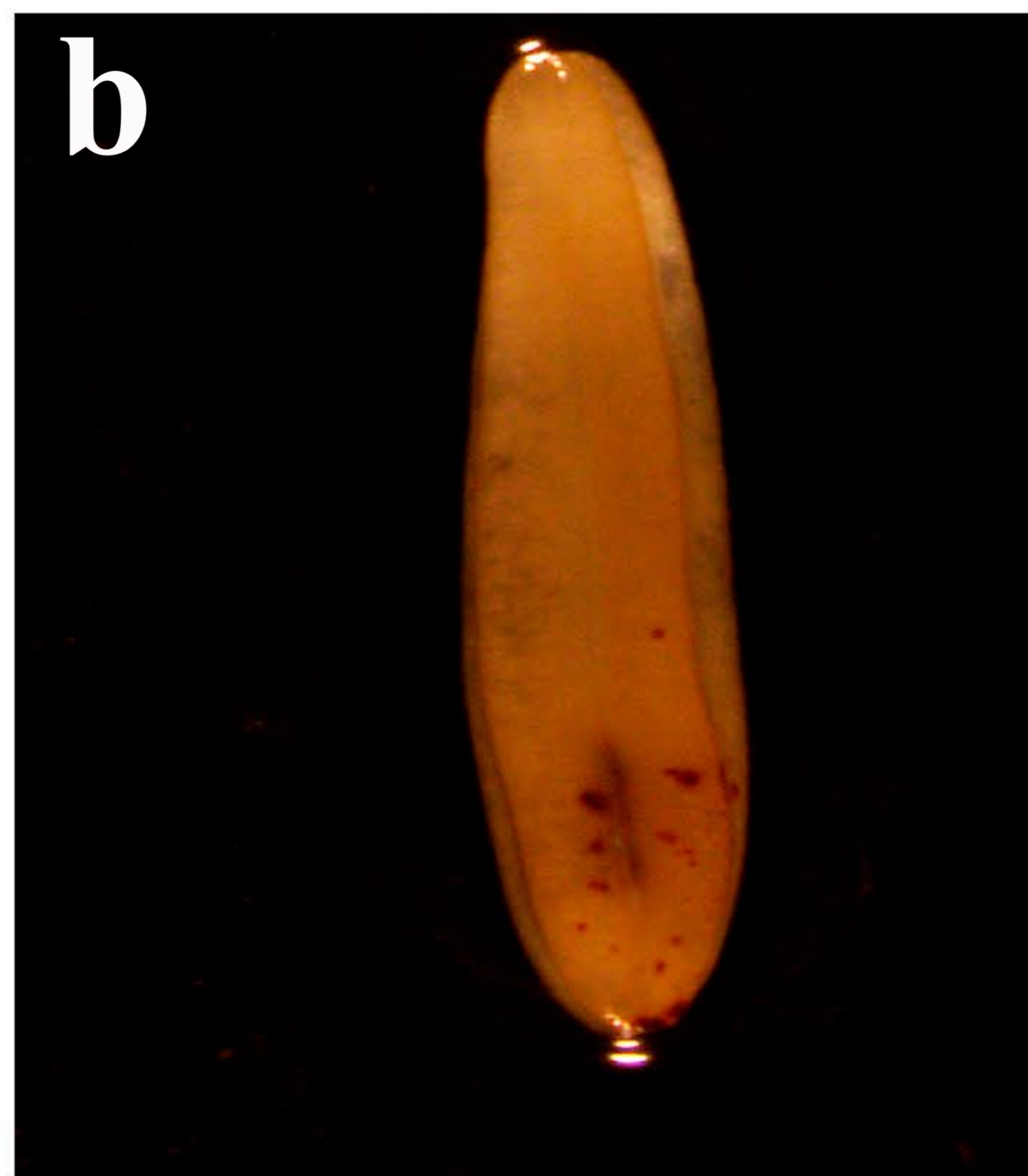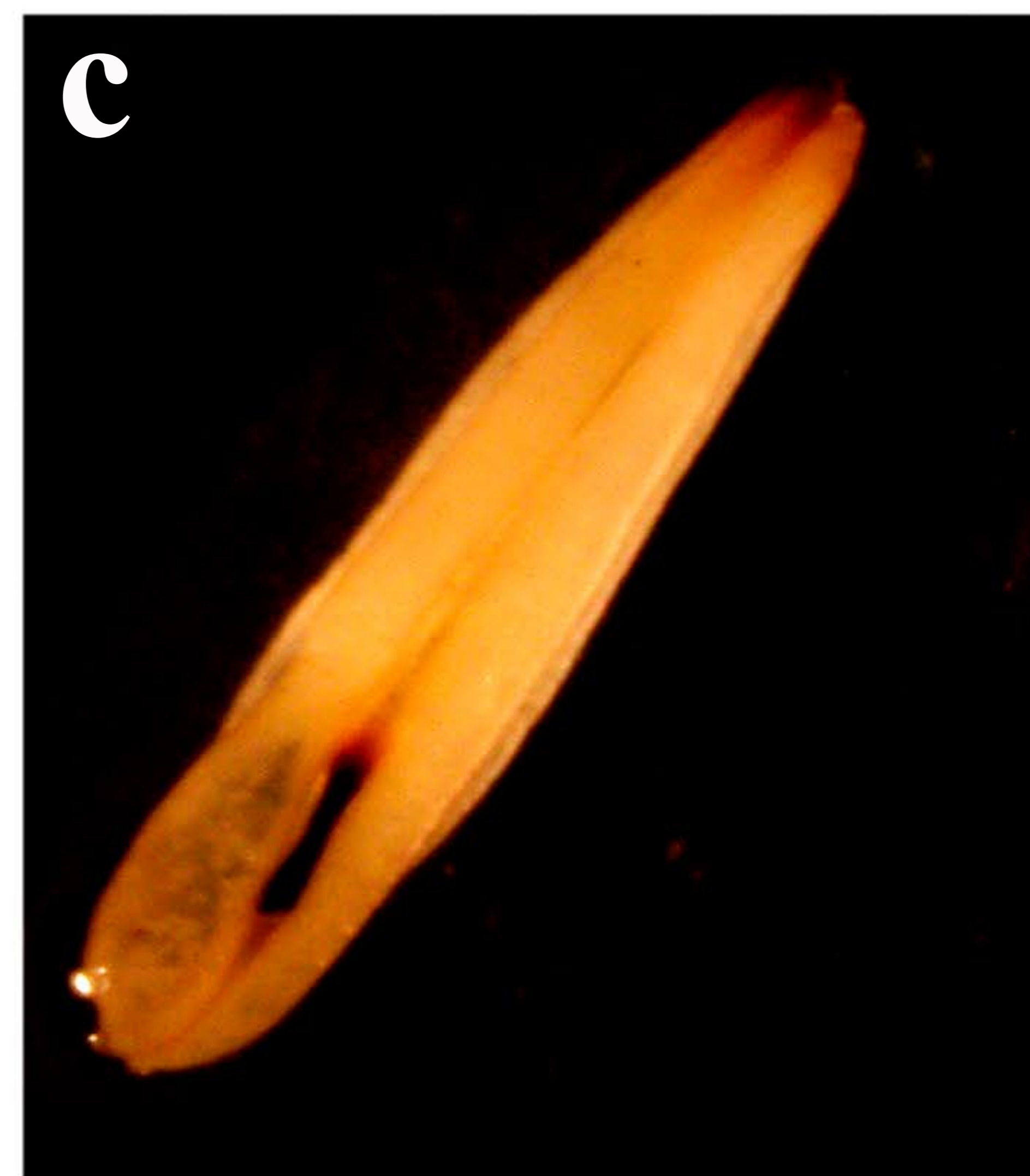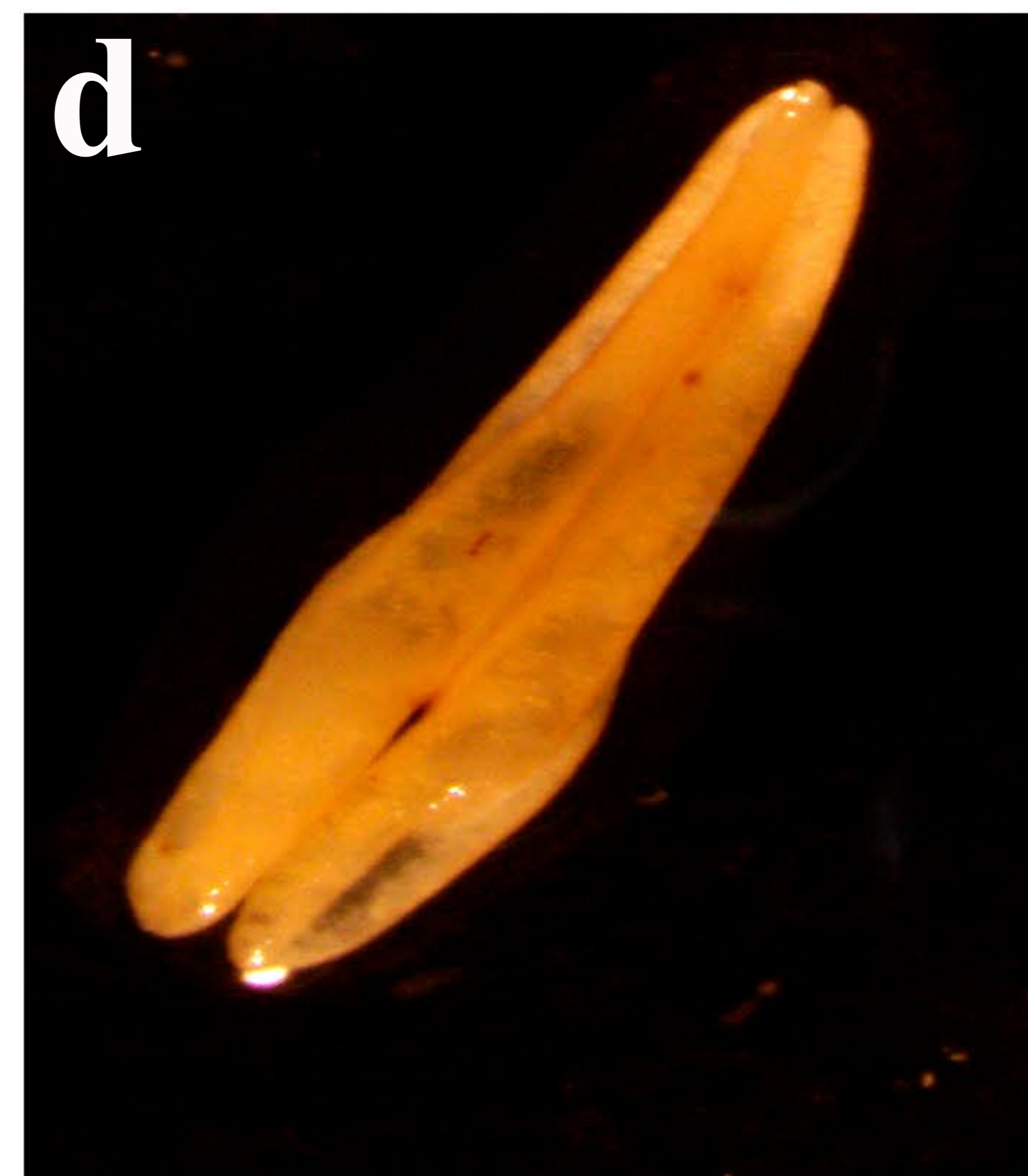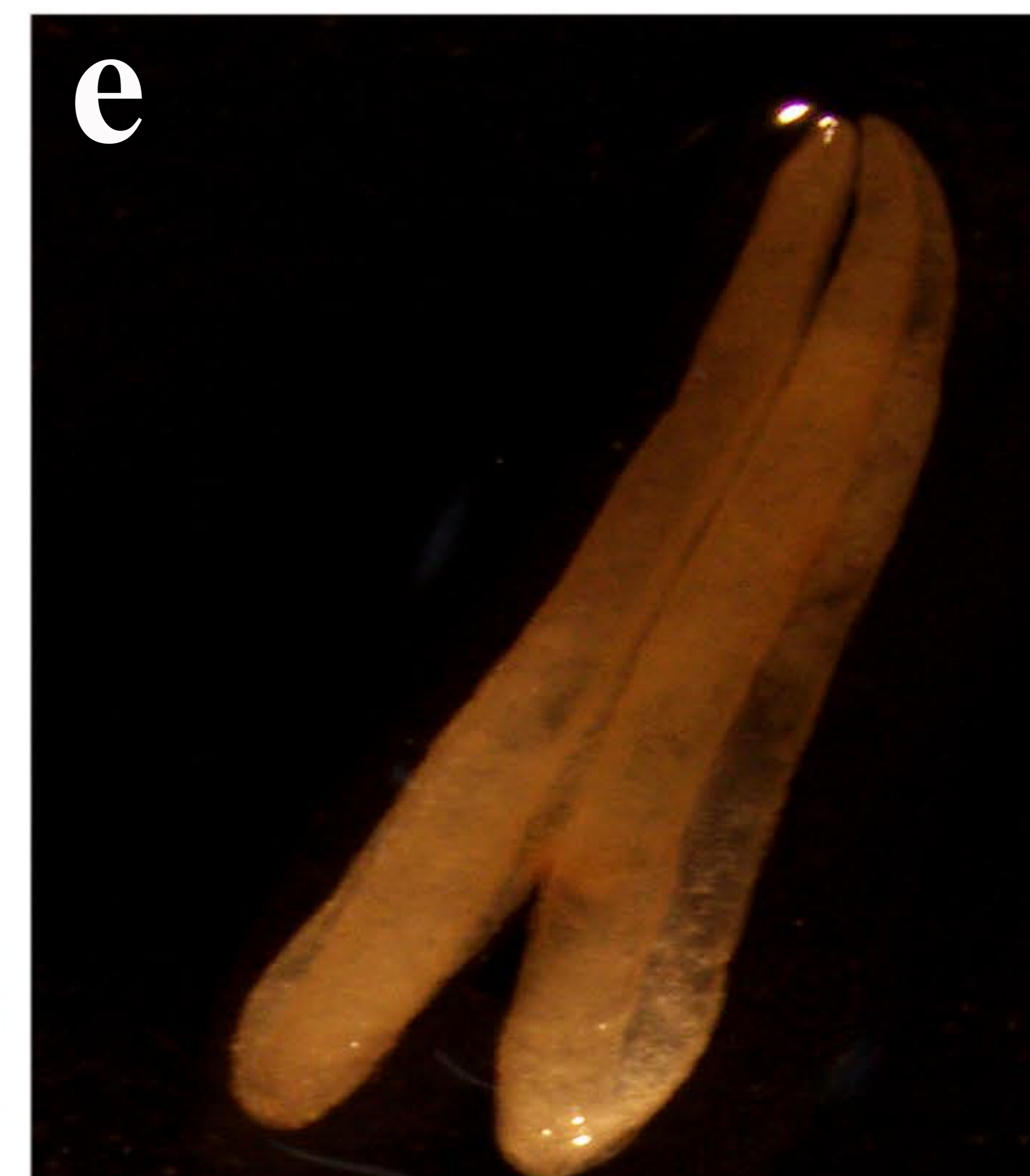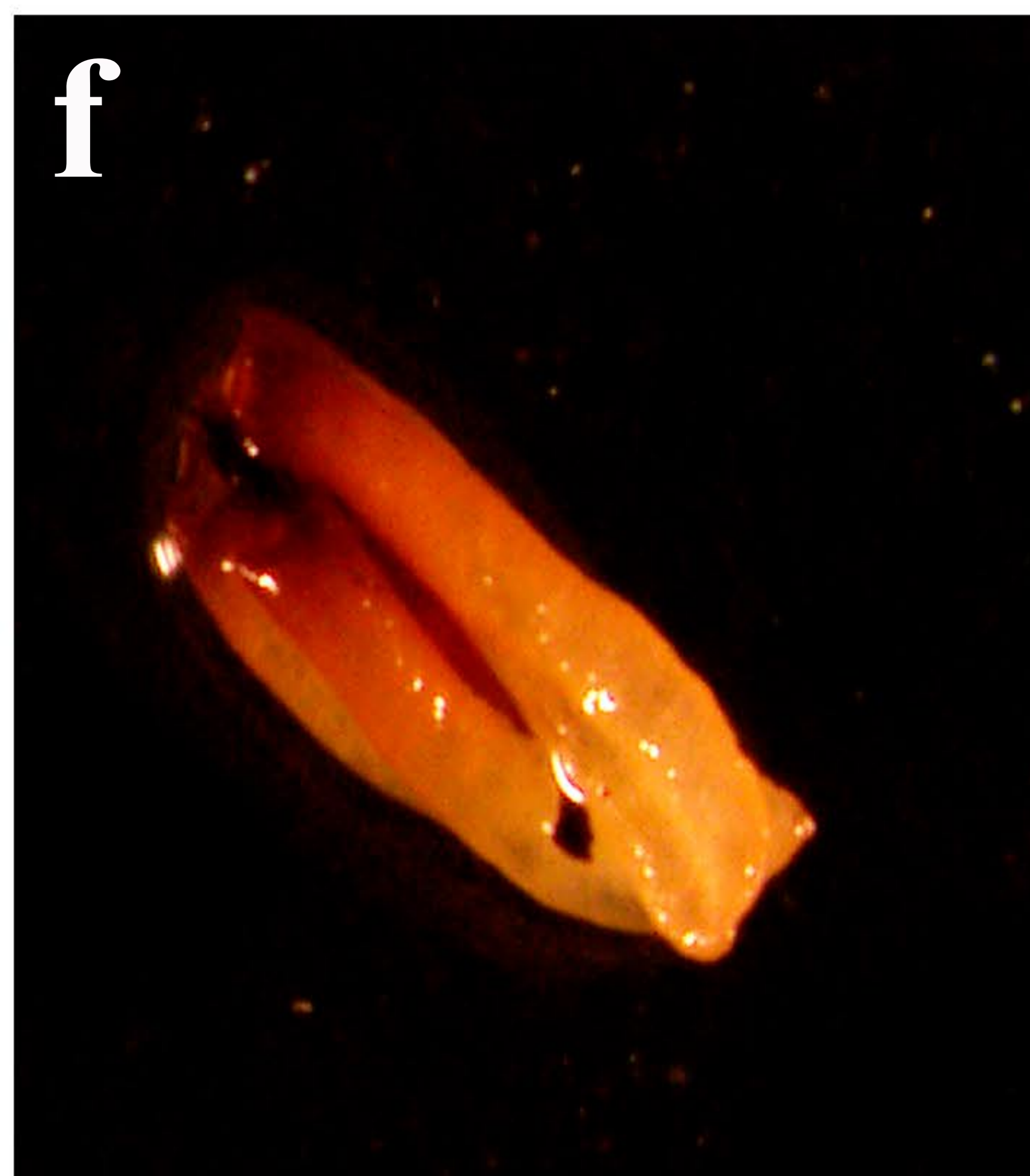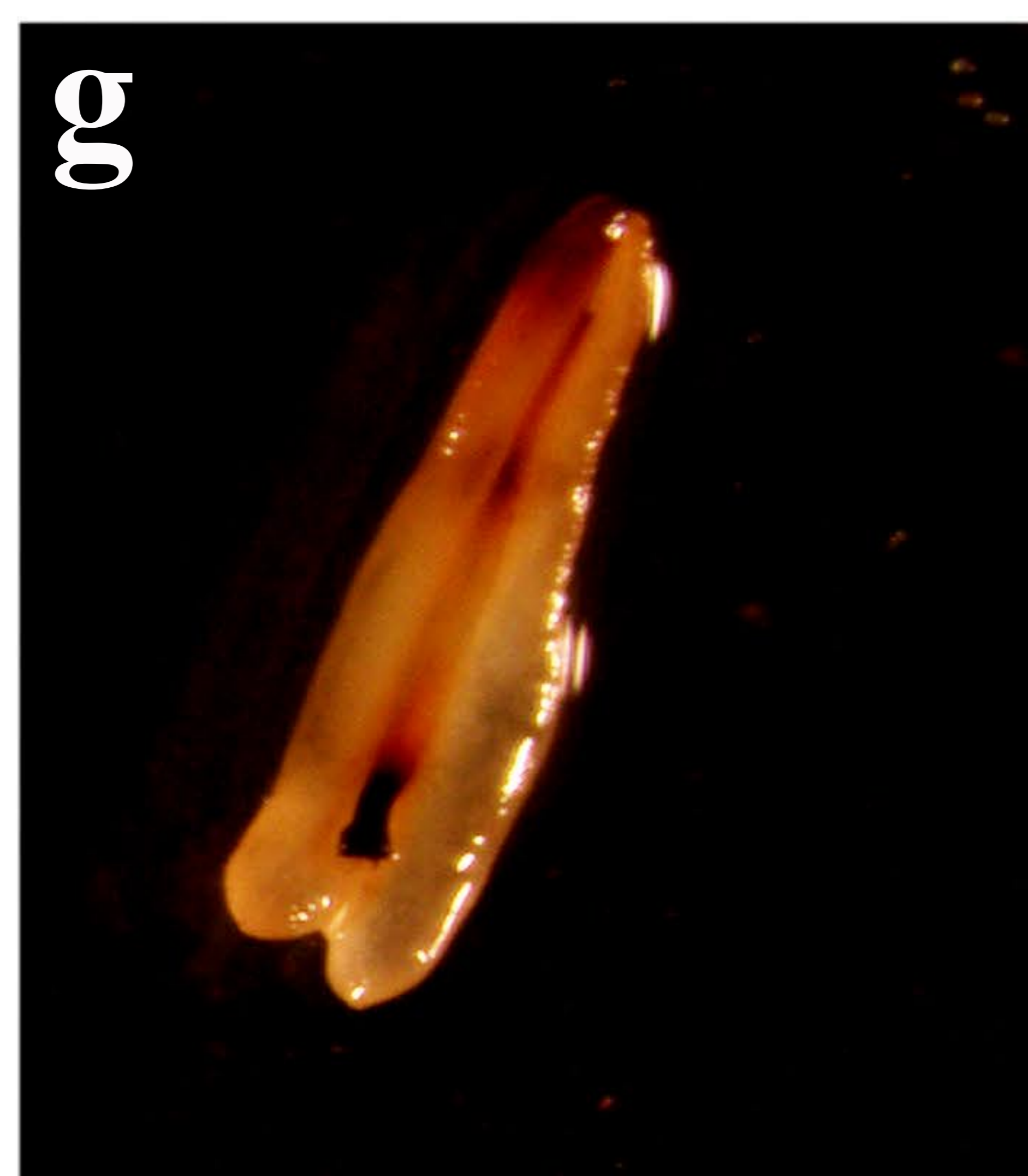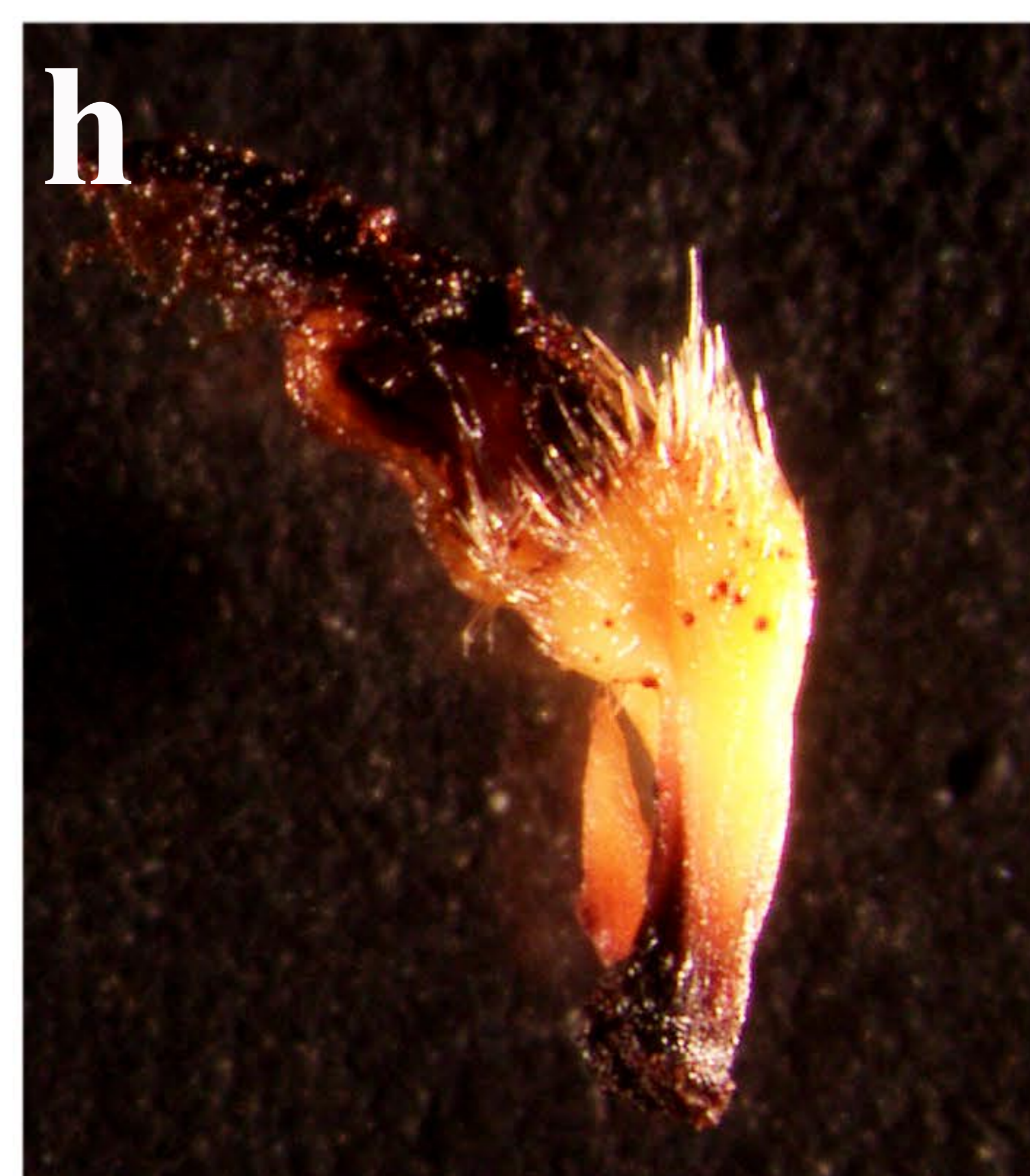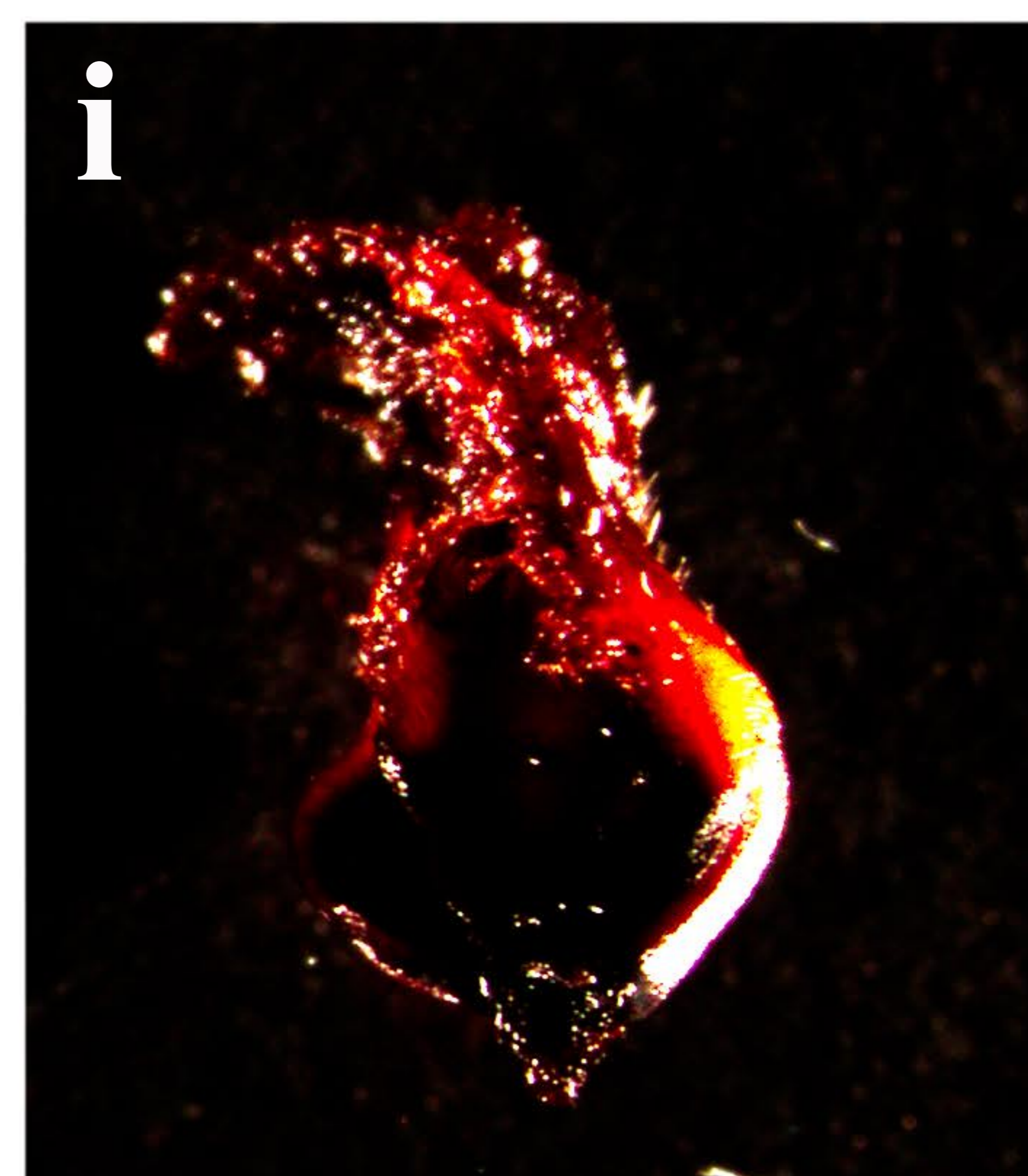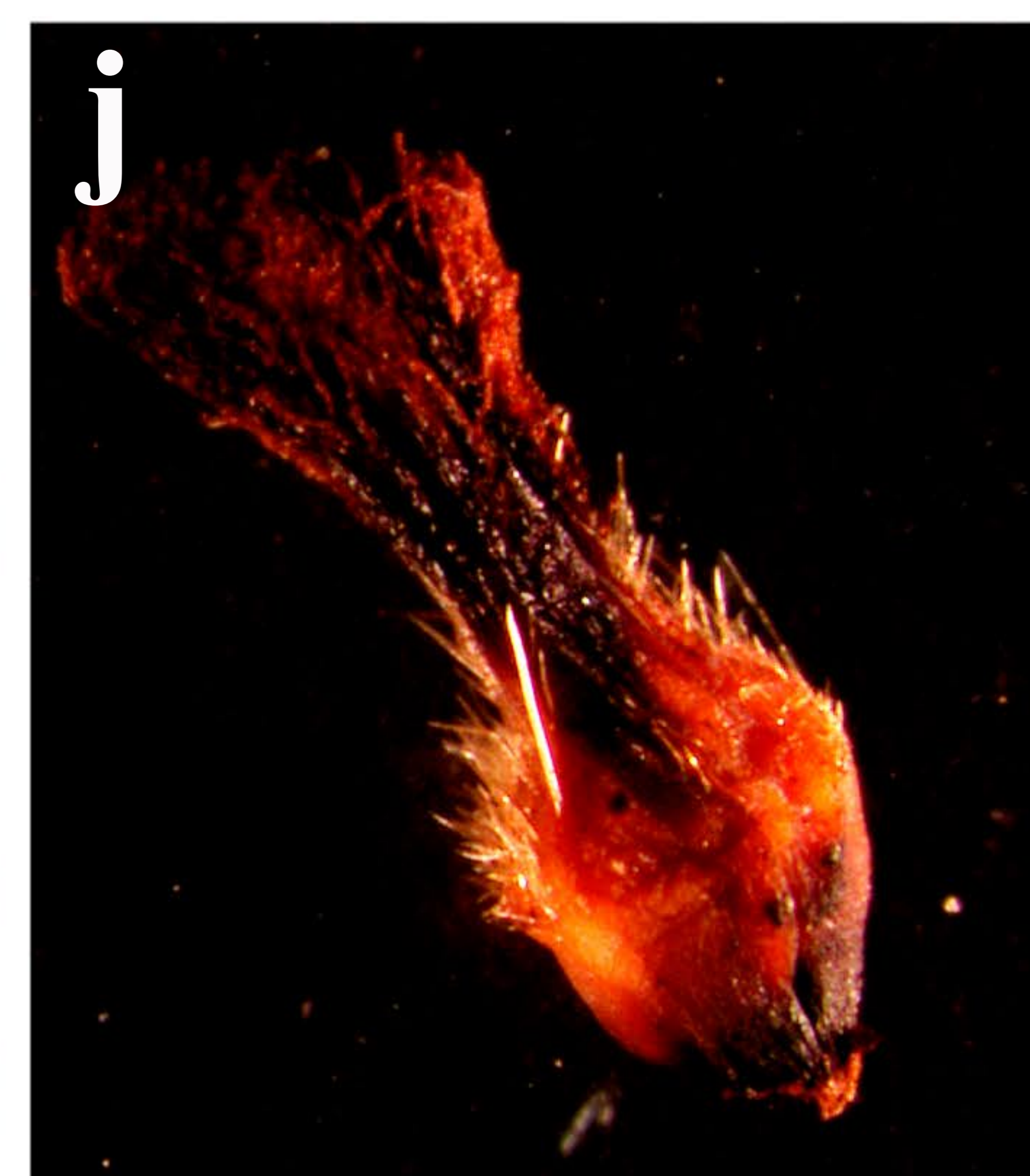

Supplement: Supplementary file 7 — Additional file 7: Figure S3: Stamens of plants stained with DAB to monitor H2O2 accumulation after 24 h in 303B (a-e) and C303A (f-j). Scale bars represent 200 μm. [file 12864_2020_6450_MOESM7_ESM.pdf]

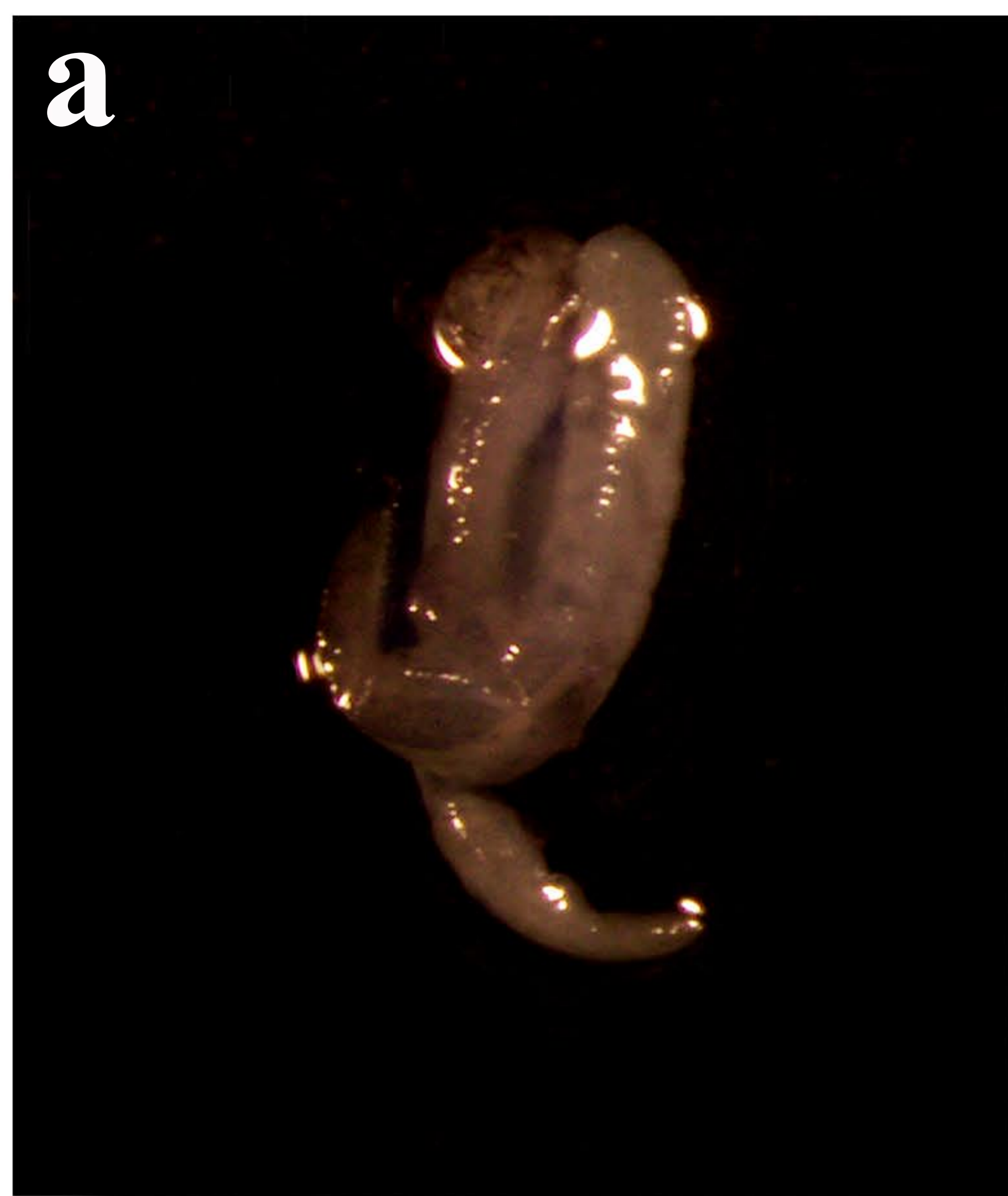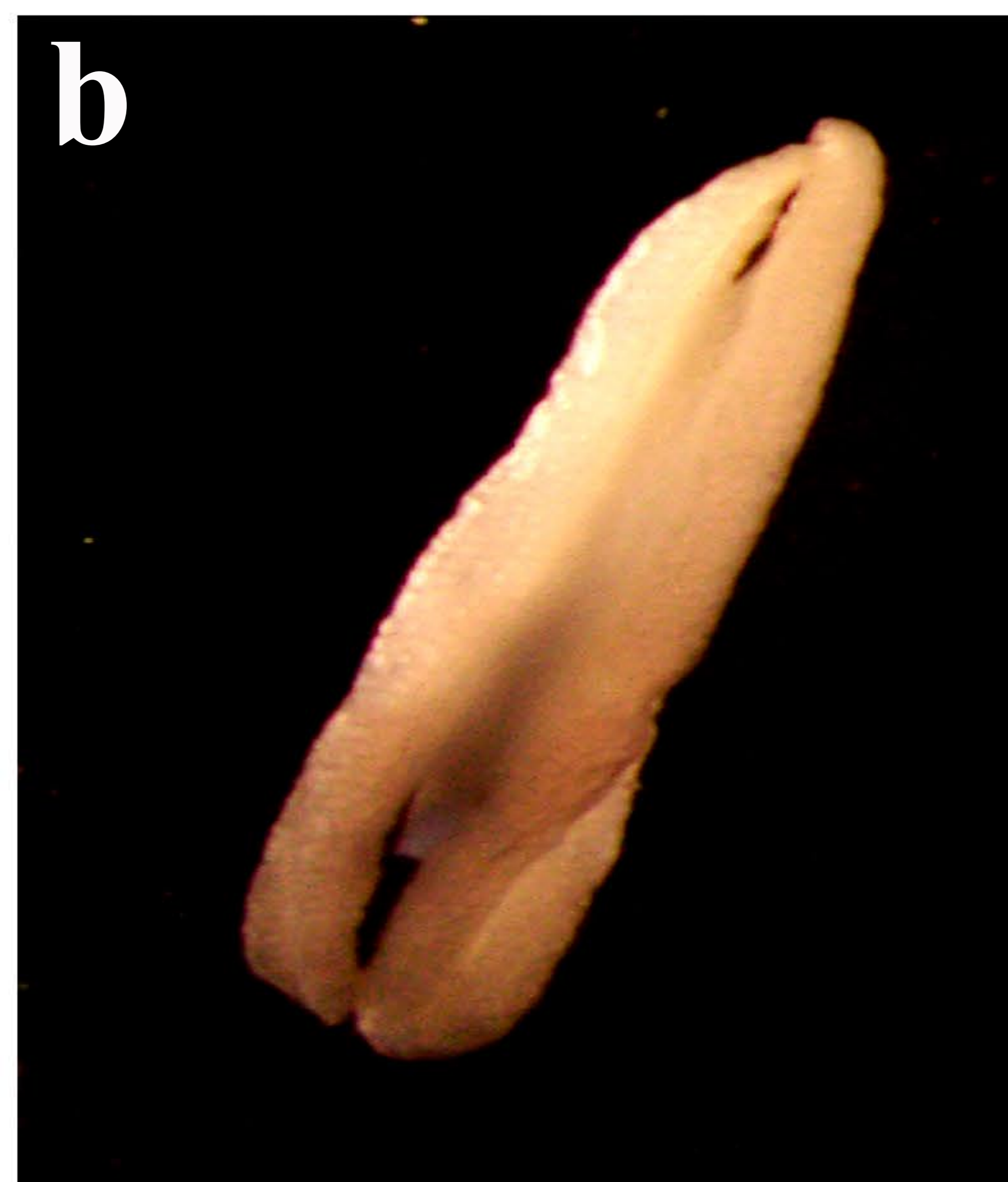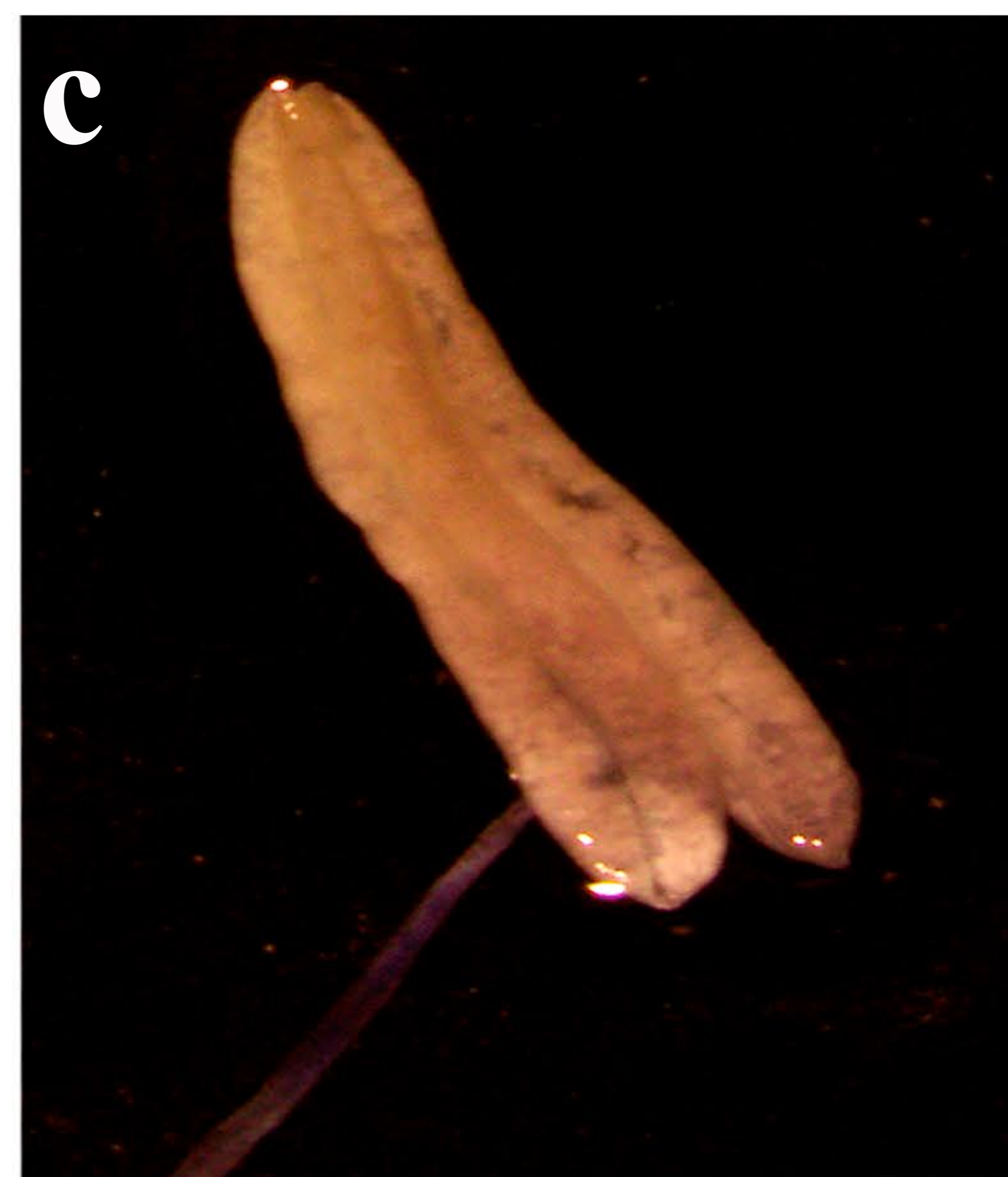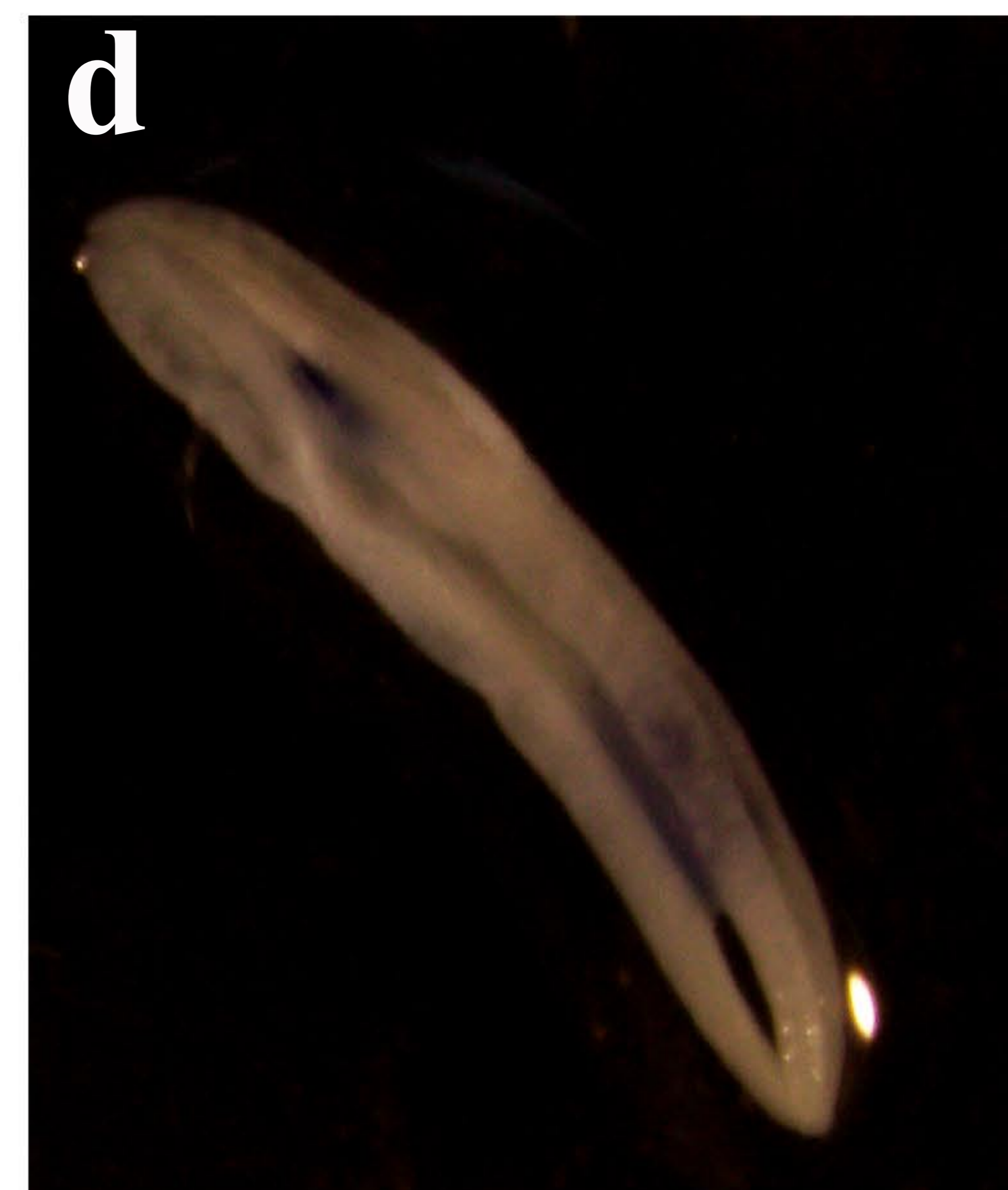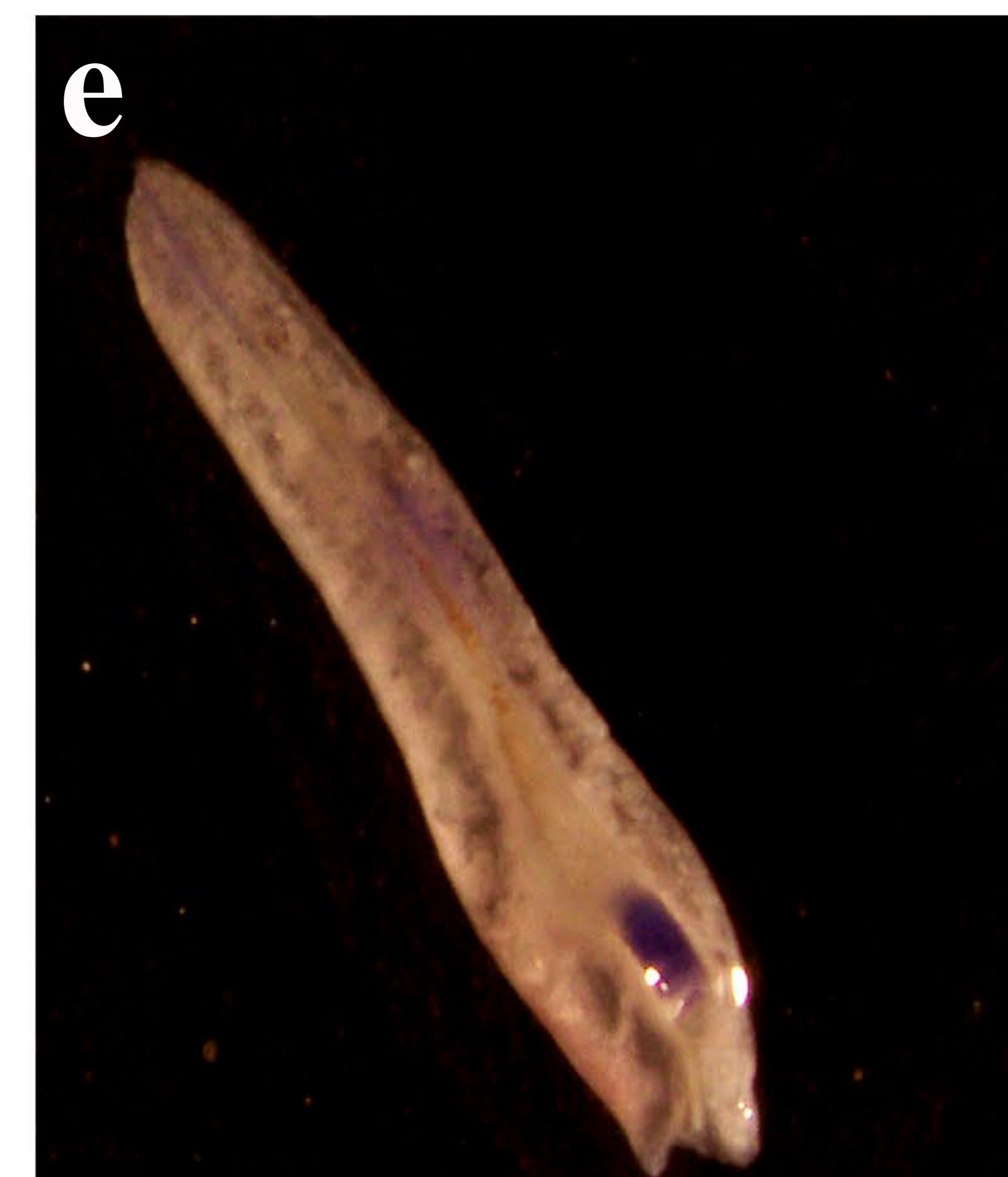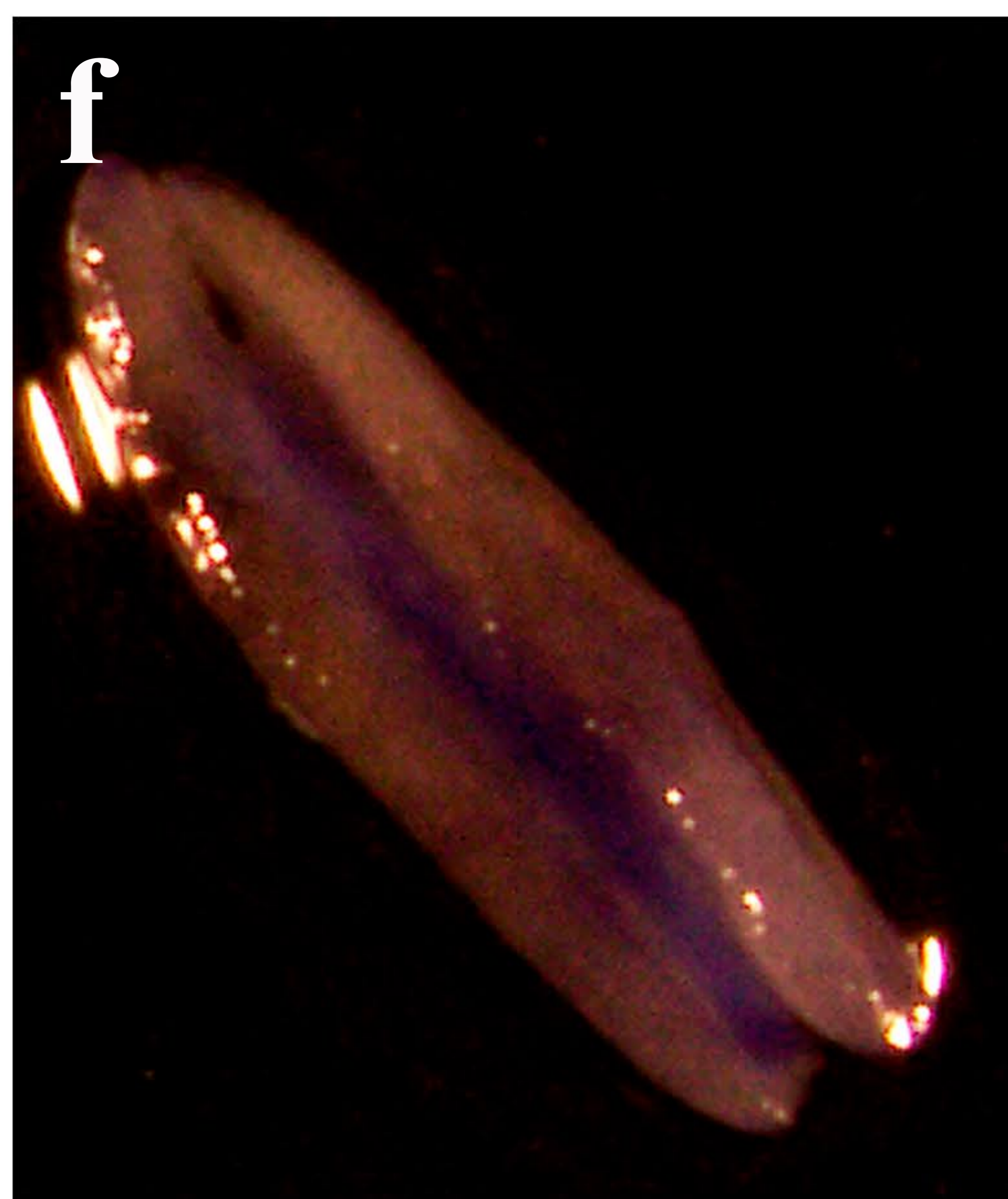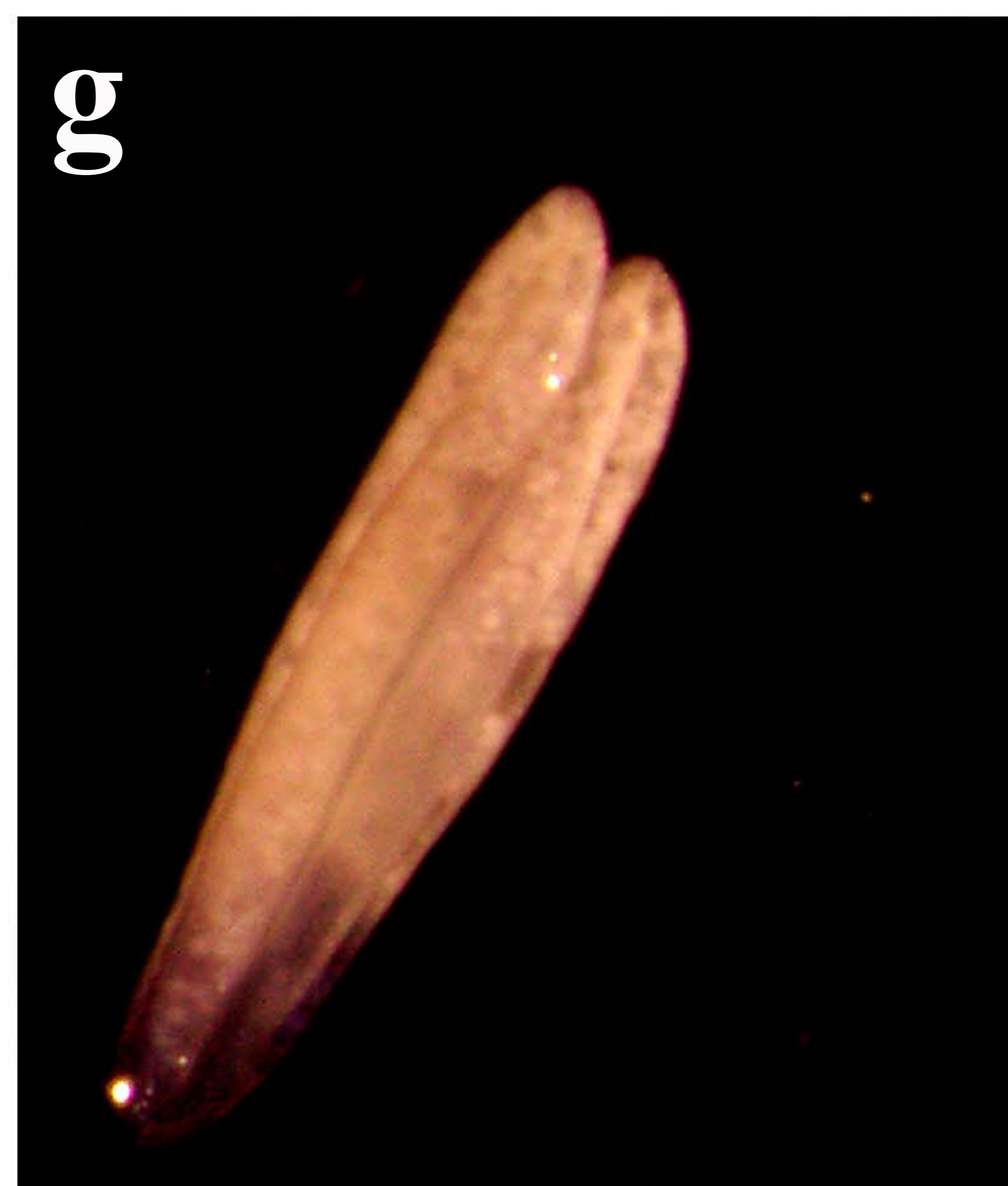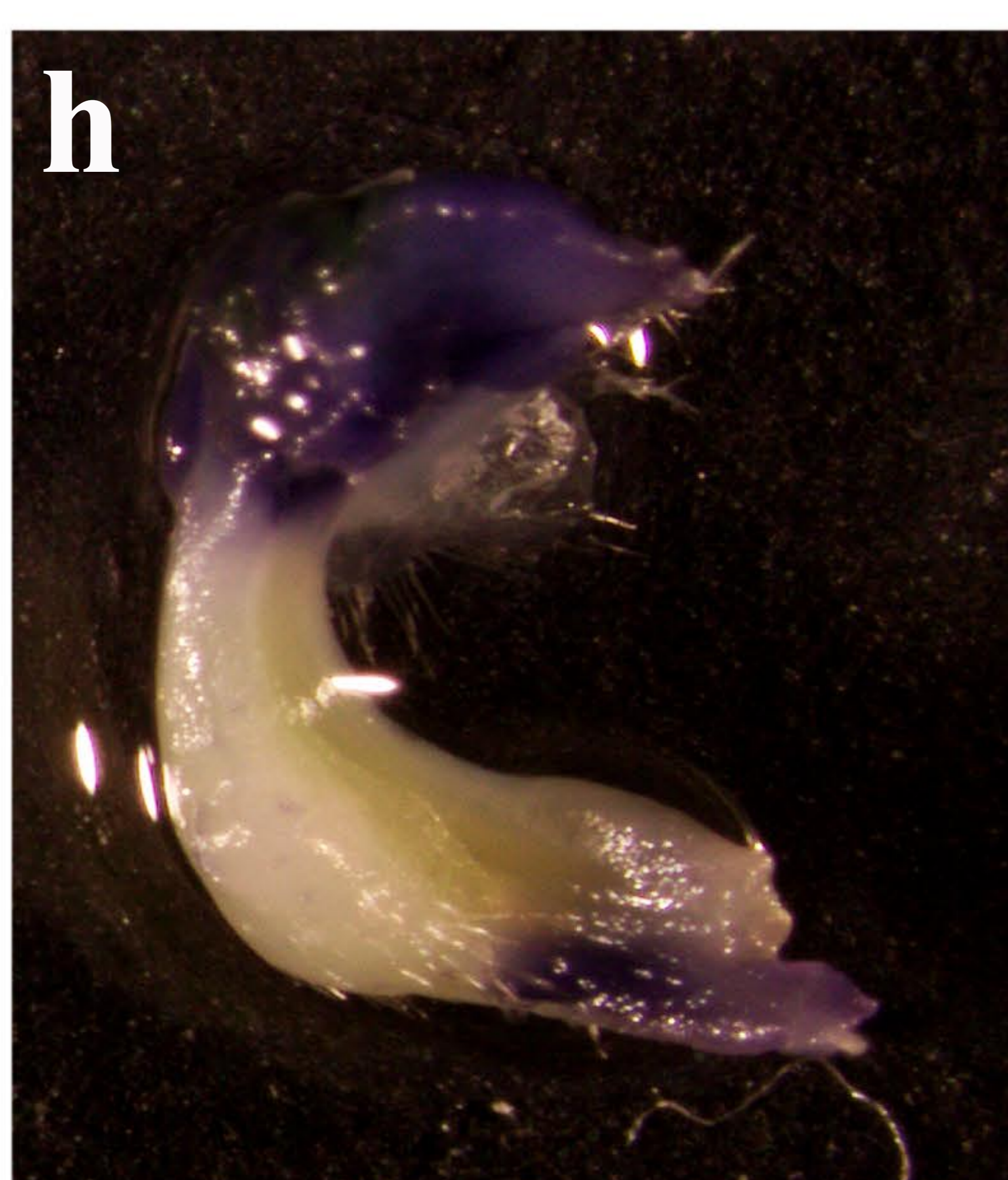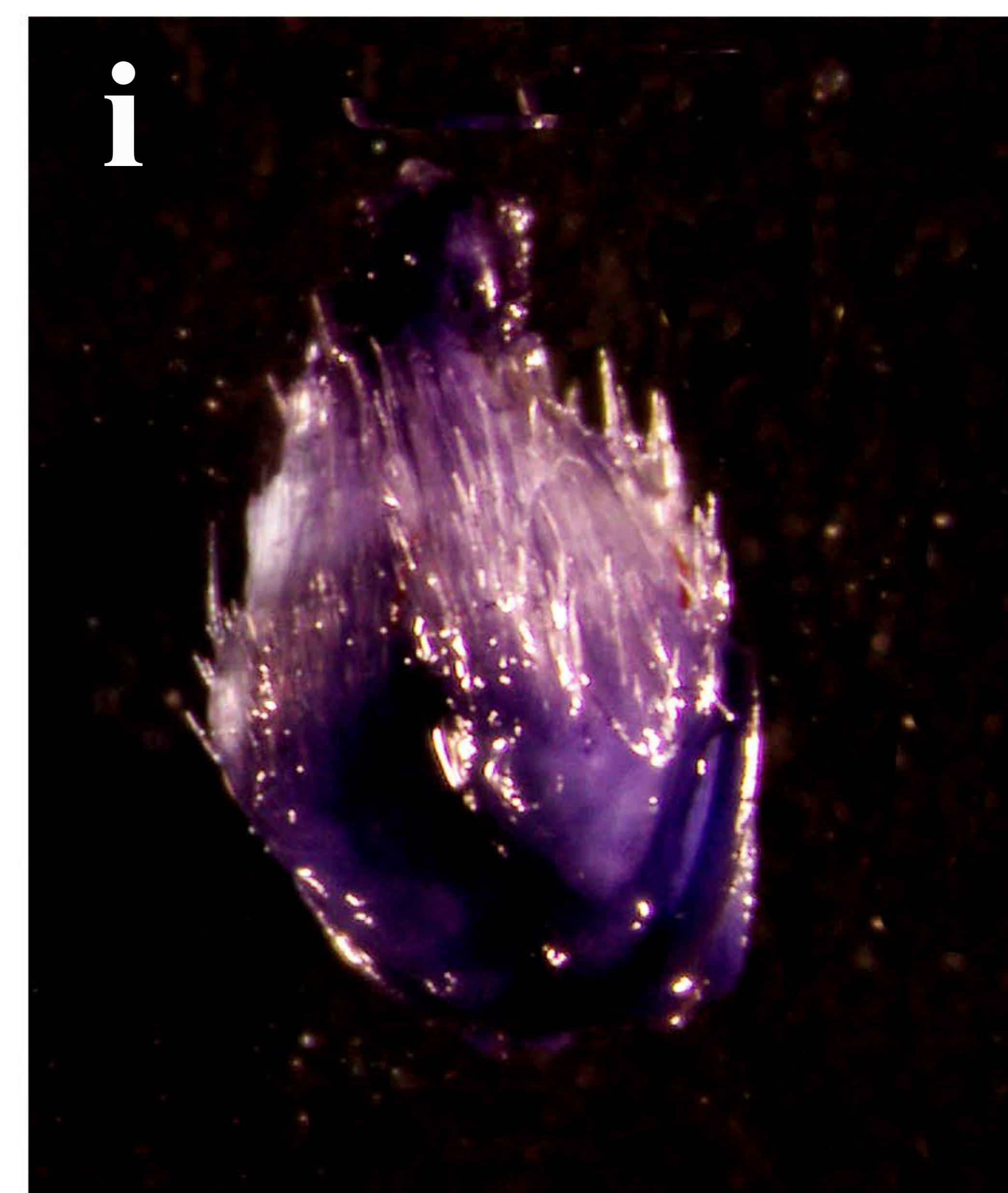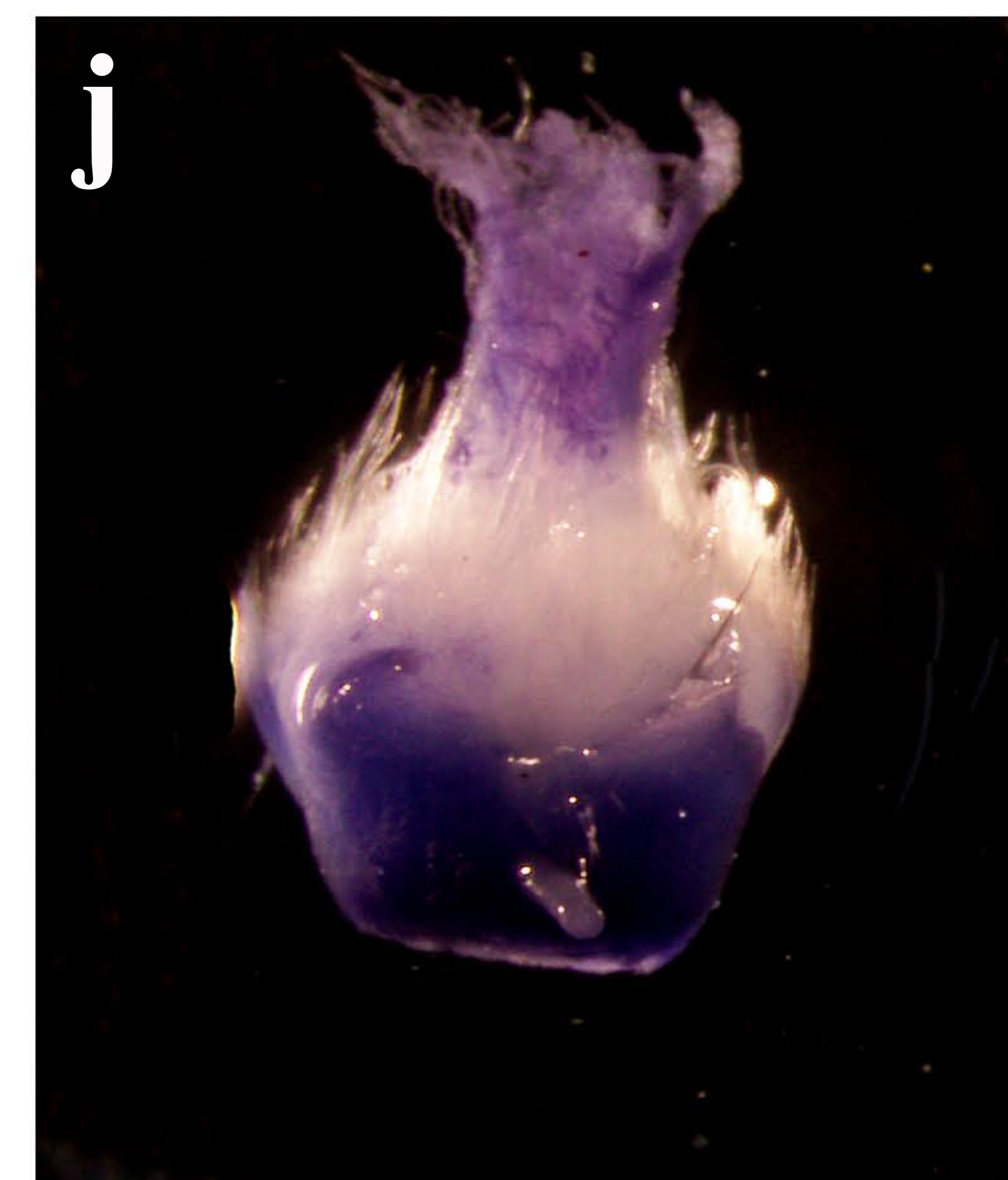

Supplement: Supplementary file 8 — Additional file 8: Figure S4: Stamens of plants stained with NBT to monitor O2− accumulation after 1 h in 303B (a-e) and C303A (f-j). Scale bars represent 200 μm. [file 12864_2020_6450_MOESM8_ESM.pdf]
